# miR-142 favors naïve B cell residence in peripheral lymph nodes

**DOI:** 10.3389/fimmu.2022.847415

**Published:** 2022-11-10

**Authors:** Magdalena Hagen, Tirtha Chakraborty, William J. Olson, Martin Heitz, Natascha Hermann-Kleiter, Janine Kimpel, Brigitte Jenewein, Johanna Pertoll, Verena Labi, Klaus Rajewsky, Emmanuel Derudder

**Affiliations:** ^1^ Institute for Biomedical Aging Research, University of Innsbruck, Innsbruck, Austria; ^2^ Program in Cellular and Molecular Medicine, Children’s Hospital, and Immune Disease Institute, Harvard Medical School, Boston, MA, United States; ^3^ Vor Biopharma, Cambridge, MA, United States; ^4^ Translational Cell Genetics, Department of Pharmacology and Genetics, Medical University of Innsbruck, Innsbruck, Austria; ^5^ Institute of Virology, Department of Hygiene, Microbiology and Public Health, Medical University of Innsbruck, Innsbruck, Austria; ^6^ Institute of Developmental Immunology, Biocenter, Medical University of Innsbruck, Innsbruck, Austria; ^7^ Immune Regulation and Cancer, Max Delbrück Center for Molecular Medicine in the Helmholtz Association, Berlin, Germany

**Keywords:** miRNA, naïve, B cells, development, migration

## Abstract

B lymphocyte development proceeds through a well-ordered sequence of steps, leading to the formation of a sizeable mature B population recognizing a diversity of antigens. These latter cells are ultimately responsible for the production of antibodies upon immune challenges. The detection of threats to the organism is facilitated by the ability of naïve follicular B cells, the main subset of mature B cells in mice, to circulate between lymphoid tissues in search of their cognate antigens. miRNA-mediated fine-tuning of mRNA stability and translation participates in the optimal expression of genetic programs. This regulatory mechanism has been shown to contribute to B cell biology, although the role of individual miRNAs remains understudied. Here, we selectively inactivated the miR-142 locus in B cells. As a consequence, the mature B compartment was visibly perturbed, in agreement with work in miR-142 knockout mice. However, our strategy allowed us to identify roles for the miR-142 locus in B cell physiology obscured by the complexity of the immune phenotype in the null mutant mice. Thus, these miRNAs are necessary for the proper formation of the pre-B cell compartment during development. More remarkably, naïve follicular B cells demonstrated altered migratory properties upon conditional inactivation of the miR-142 locus. The latter mutant cells expressed reduced levels of the homing molecule CD62L. They also migrated more efficiently towards sphingosine-1-phosphate *in vitro* and displayed an increased abundance of the sphingosine-1-phosphate receptor 1, compatible with improved lymphocyte egress *in vivo*. In line with these observations, the ablation of the miR-142 locus in B cells caused a paucity of B cells in the lymph nodes. Mutant B cell accumulation in the latter tissues was also compromised upon transfer into a wild-type environment. These changes coincided with suboptimal levels of FOXO1, a positive regulator of CD62L transcription, in mutant B cells. Overall, our findings indicate contributions for the miR-142 locus in various aspects of the B cell life cycle. Notably, this locus appears to favor the establishment of the migratory behavior required for naïve follicular B cell patrolling activity.

## Introduction

Mature B cells are key players of the adaptive immunity through their ability to synthesize antibodies in response to stimulation. Several subsets have been identified within the mature B compartment in mice (1). Thus, marginal zone (MZ) B cells reside along the marginal sinuses bordering the white pulp in the spleen and are first responders to blood-borne pathogens (2). B1 cells are prominent contributors to the production of natural antibodies and predominate in the pleural and peritoneal cavities (3). Finally, follicular (Fo) B cells constitute the most substantial mature subset (1). These latter cells, named after their localization in follicles of secondary lymphoid tissues, are the main players in T-dependent responses. Mature B cells develop from committed progenitors present in the fetal liver and adult bone marrow (BM) in a precisely organized succession of stages (4).

A fundamental aspect of naïve Fo B cell physiology is their ability to migrate between lymphoid tissues to monitor for the presence of their cognate antigens. Thus, these cells enter and leave lymph nodes (LNs) following highly ordered processes. In brief, naïve Fo B cells in the blood are slowed down at high endothelial venules (HEVs) in the LNs (5–7). The latter tethering and rolling step depends on the interaction between the adhesion molecule L-selectin (CD62L) expressed by B cells and specific carbohydrate moieties (sialyl Lewis X) attached to surface proteins on endothelial cells. Subsequently, signals from the CCR7 and CXCR4 chemokine receptors, upon detection of their respective ligands CCL19/21 and CXCL12 at the HEVs, lead to the firm adhesion of Fo B cells by primarily promoting the binding of the integrin LFA-1 to ICAM molecules on the endothelium. Eventually, these cells transmigrate through the HEVs to the inside of the LNs. In the absence of antigen detection, Fo B cells move back to the circulation following a sphingosine-1-phosphate (S1P) gradient. B cell egress is mediated by signaling from the S1P receptor 1 (S1PR1) *in vivo* (6, 8).

The generation and function of B cells rely on the proper execution of genetic programs. Appropriate gene expression is controlled by the activity of miRNAs, which regulate mRNA stability and translation (9). An essential role for miRNAs in B cell physiology is illustrated by the block in development observed in the absence of Dicer and Drosha (10, 11), two enzymes involved in the biogenesis of these RNAs (9). In addition, several miRNAs have been shown to regulate various stages of the B cell life cycle, although the list is limited. Thus, the miR17∼92 cluster, miR-150 and miR-155 contribute to precursor B cell formation, the generation of B1 cells and B cell responses to T-dependent antigens, respectively (12–14).

The miR-142 locus encodes three mature miRNAs in mice: miR-142-5p, miR-142-3p, and the lesser-known miR-142b (miRbase.org). This locus is particularly well expressed in hematopoietic cells and appears critical for the homeostasis of the immune system (15). Among others, defects in the physiology of neutrophils, dendritic cells, type 1 innate lymphoid cells and T cells are observed upon germline inactivation of the miR-142 locus in mice (16–20). Remarkably, mature B cellularity has been reported increased in the spleen of miR-142-null mice due to an accumulation of MZ B cells (17, 19). The enhanced expression of the pro-survival receptor, and miR-142-3p target, BAFFR appears to drive this aberrant expansion (17). In addition, mature B1 cells are absent in the peritoneal cavity of such mutant mice (17). The differentiation of miR-142-deficient B cells into plasma cells appears also compromised upon stimulation (17). However, the phenotypic complexity of the null mice precludes the proper evaluation of miR-142 B cell-intrinsic functions.

Here, we employed a conditional gene targeting strategy to inactivate the miR-142 locus selectively in B cells. This approach permitted the identification of previously unnoticed B cell functions for these miRNAs. Specifically, the miR-142 locus appears to play roles in ensuring a full BM precursor B compartment and in the control of naïve Fo B cell migratory properties. Unexpectedly, an accumulation of splenic B cells was not observed in our Mb1-cre^+^ miR-142^fl^ mice, suggesting a possible contribution for (an) extrinsic signal(s) in the expansion of mature B cells in the null mutant mice.

## Materials and methods

### Generation of the miR-142^fl^ allele

677 bp of C57BL/6 mouse chromosome 11 containing the miR-142 precursors were amplified and cloned into an AscI site in-between the two loxP sites of the targeting vector. In addition, 2.2 kb (5’) and 3.5 kb (3’) of flanking arms of homology were inserted in BglII - NotI and HindIII – XhoI, respectively. The construct was then introduced into C57BL/6 embryonic stem cells (Artemis). Targeted ES cells were selected for Neomycin-resistance and correct genomic insertion of the floxed miR-142 fragment was evaluated using a Southern blotting strategy. A properly targeted embryonic stem cell clone was injected into C57BL/6 albino blastocysts and the obtained chimera crossed with C57BL/6 mice. The progeny was then bred to FLPe-deleter mice to eliminate the FRT-flanked Neomycin resistance gene (21), ultimately generating the miR-142^fl^ mouse strain. Finally, the LoxP-flanked miR-142 fragment was amplified from the genome of a miR-142^fl^ mouse and sequenced to validate the targeting of the locus.

### Mice

Mb1-cre, R26-Stop^fl^-hCD2 and deleter-cre mice have been previously described (22–24). The Mb1-cre strain was obtained from The Jackson Laboratory (stock: 020505). miR-142-deficient mice were generated by breeding miR-142^fl^ with deleter-cre animals and subsequently intercrossing the offspring carrying a deleted miR-142 allele. Mice were on the C57BL/6 background and kept under specific pathogen-free conditions. Animals were analyzed between 7 and 24 weeks.

Although no obvious segregation of the control genotypes could be noticed in the data, we grouped these animals for clarity as follows (+, f and Δ indicate wild-type, floxed and deleted alleles): Mb1-cre^–^: miR-142^f/+^, miR-142^f/f^ and miR-142^f/Δ^. Mb1-cre^+^: Mb1-cre^+^ miR-142^f/+^, Mb1-cre^+^ miR-142^+/Δ^ and Mb1-cre^+^. The group of mutant mice (Mb1-cre^+^ miR142^fl^) comprises Mb1-cre^+^ miR-142^f/f^ and Mb1-cre^+^ miR-142^f/Δ^. The presence of deleted alleles in mice stemmed from the sporadic activity of Mb1-cre in germ cells (25). Mice could additionally bear the R26-Stop^fl^-hCD2 reporter gene.

Mice of both sexes were included in the present work. When analyzed separately, male (n=5-16) and female (n= 4-5) mutant mice tended to show similar alterations in pre- as well as mature B cellularity, frequency of lymph node B cells and S1P-mediated chemotaxis (data not shown). These observations suggest the absence of a strong gender bias induced by the inactivation of the miR-142 locus in B cells.

### Immunofluorescence microscopy

Pieces of spleens were embedded in Tissue-Tek OCT (Sakura Finetek) and frozen at -80°C. 7 μm-thick splenic sections were cut on a Microm HM 500 OM cryotome and laid on polylysine-coated slides (Thermo Scientific). Samples were fixed in acetone for 5 min, air dried, then washed twice in PBS (136.8 mM NaCl, 2.7 mM KCl, 10 mM Na_2_HPO_4_·7 H_2_O, 1.76 mM KH_2_PO_4_; Carl Roth) and twice in 0.1% Tween 20 PBS. Slides were next incubated in a blocking solution of 5% skim milk (Sigma-Aldrich) in PBS for 30 min at room temperature, followed by two washes in Tween-PBS. Sections were subsequently stained with B220-PerCP-Cy5.5 (RA3-6B2; Biolegend) and CD169-Bv421 (3D6.112; Biolegend) in blocking buffer, first for 1-2h at room temperature and next at 4°C overnight in a humidifying chamber, then washed twice in Tween-PBS and once in PBS. Finally, cover slips were fixed to the slides using Fluoromount G (ThermoFischer). Data were acquired on a confocal microscope Cell Voyager CV1000 (Yokogawa) using the CV1000 software and processed with the Fiji software (26).

Perimeters of B220^+^ B cell populations contiguous to CD169^+^ macrophage rings, as well as the circumference of the latter rings, were manually outlined in confocal immunofluorescence images using the Fiji software to estimate white pulp and marginal zone areas (in pixels^2^). An index of circularity (4π x Area/Perimeter^2^) was also computed, with the maximum value of 1 representing a true circle. In addition, the surfaces occupied by B cells within the two defined areas were calculated using the Fiji software based on B220 fluorescence, selecting the signal intensity above background.

### Flow cytometry

Single cell suspensions of spleen, bone marrow (1 hind leg), peripheral lymph nodes as well as peritoneal cavity were prepared in Dulbecco’s PBS 1x (Sigma-Aldrich) with 2% fetal bovine serum (ThermoFisher). In addition, spleen and bone marrow preparations were treated with Gey’s solution to remove red blood cells. Cells were stained with the subsequent antibodies conjugated to APC, biotin, Bv421, Bv510, Bv605, Bv785, FITC, PE, PE-Cy7 or PerCP-Cy5.5: B220 (RA3-6B2), CD1d (1B1), CD11b (M1/70), CD19 (6D5), CD21 (7E9), CD23 (B3B4), CD25 (PC61), CD3ε (145-2C11), CD5 (53-7.3), CD62L (REA828), CD93 (AA4.1), c-Kit (2B8), hCD2 (RPA-2.10, TS1/8), IgD (11-26c.2a), IgM (Fab fragment and RMM-1), Itgα4 (CD49d; R1-2), Itgβ1 (CD29; HMβ1-1), LFA-1 (CD11a/CD18; H155-78), Ly6G (1A8), S1PR1 (713412) and TCRβ (H57-597) obtained from Biolegend, Jackson ImmunoResearch, Miltenyi, R&D systems and ThermoFisher. When required, samples were first stained with biotinylated antibodies followed by a second step with mixes including streptavidin coupled to an aforementioned fluorochrome and other labelled antibodies. Data were acquired on FACSCanto II and LSRFortessa flow cytometers (BD Biosciences) and analyzed using the FlowJo software (BD Biosciences).

### B cell isolation

Splenic B cells were enriched by magnetic depletion, removing non-B cells using the following antibodies coupled to biotin: CD11b (M1/70), CD11c (N418), CD4 (GK1.5), CD5 (53-7.3), CD8α (53-6.7), Gr1 (RB6-8C5), NK1.1 (PK136) as well as Ter119 (TER-119) (Biolegend), and the MagniSort Streptavidin Negative Selection Beads according to the manufacturer’s instructions (ThermoFisher). Alternatively, splenic CD19^+^B220^+^CD93^–^IgM^+^CD1d^+^ Fo B cells were FACS-isolated on a FACSAriaIII (BD Biosciences).

### Chemotaxis assay

5x10^6^ splenocytes/ml were serum-starved for 2h at 37°C and 5% CO_2_ in high glucose DMEM medium (Sigma-Aldrich) supplemented with 0.5% fatty acid-free BSA (Carl Roth), 2 mM L-glutamine (Sigma-Aldrich), 10 mM HEPES (Lonza), 100 U/ml penicillin and 100 μg/ml streptomycin (Sigma-Aldrich). Cell migration was assessed by placing 5 μm transwell inserts (Sarstedt) in 24-well plates. 600 μl medium containing or not 1 μg/ml mouse CCL21 (Peprotech), 30 or 100 ng/ml mouse CXCL12/SDF1α (Peprotech) and, 20 or 100 nM d18:1 sphingosine-1-phosphate (Sigma-Aldrich) (27–30), were supplied to the bottom chambers and 5x10^5^ splenocytes were added to the top chambers. In addition, 5x10^5^ cells were seeded in a well filled with 600 μl of medium without transwell insert (input). Plates were incubated for 3h at 37°C and 5% CO_2_. Cells collected from the bottom chambers and the input wells were subsequently stained to identify CD23^+^ and CD23^lo/–^ CD19^+^B220^+^CD93^–^IgM^+^CD1d^+^ Fo as well as CD19^+^B220^+^CD93^–^IgM^hi^CD1d^hi^ MZ B cells in flow cytometry. Events were recorded for 90s at medium speed on a LSRFortessa (BD Biosciences). B cells that migrated to the lower chambers were expressed as proportions of the inputs as follows: (events of B cell subset in the lower chamber/events of B cell subset in the input) x 100. A minimum threshold of 20 events/given population detected in the bottom chamber upon treatments was applied for inclusion in the analysis.

### Adoptive transfer

Splenocytes isolated from Mb1-cre^+^ control and mutant mice were labelled for 10 min at 37°C with Cell Proliferation Dye eFluor450 and eFluor670 (10 μM final), respectively, following the provided protocol (ThermoFisher). Cell suspensions were counted and equal numbers of labelled splenocytes (in Dulbecco’s PBS 1x) from pairs of control and mutant donor mice were mixed. Recipient animals (two for each donor mix) were injected intravenously with 200 μl (14 - 20x10^6^ cells) of the cell preparations using insulin syringes. After 2h, the spleen and peripheral lymph nodes of the recipient mice were collected for flow cytometry.

From these data, the B (% of CD19^+^ cells)/T (% of CD5^+^ cells) ratios within Mb1-cre^+^ and Mb1-cre^+^ miR142^fl^ donor cells in the spleens and peripheral lymph nodes of the recipients were determined and normalized to the B/T ratios in the respective donor mixes. In addition, normalized donor mutant/control ratios within IgM^+/hi^CD21^+/hi^CD93^–^ mature B cells were similarly calculated.

Recipient mice were of the following genotypes: wild type, miR-142^f/+^ and miR-142^f/+^ R26-Stop^fl^-hCD2.

### Western blotting

2x10^6^ magnetically-isolated splenic B cells were lysed in whole cell extraction buffer: 25 mM HEPES (Sigma-Aldrich), 0.3 M NaCl (Carl Roth), 1.5 mM MgCl_2_ (Carl Roth), 0.2 mM EDTA pH 8.0 (Sigma-Aldrich), 0.5% Triton X-100 (Sigma-Aldrich), 10 mM NaF (Sigma-Aldrich), 10 mM Na-pyrophosphate (Sigma-Aldrich), 100 μM Na-o-vanadate (Sigma-Aldrich), 2 mM DTT (Sigma-Aldrich), supplemented with protease inhibitors (cOmplete Protease Inhibitor Cocktail, Roche). 30-50 μg of proteins were separated by SDS-PAGE (31) and transferred onto PVDF membranes (Merck). Membranes were subsequently probed with anti-FOXO1 (C29H4, Cell Signaling Technology) and anti-HSP90 (F-8, Santa Cruz Biotechnology) primary antibodies, goat anti-mouse as well as anti-rabbit IgG secondary antibodies conjugated to HRP (Bio-Rad), and signals visualized using ECL prime reagent (GE Healthcare). Data were recorded on Chemidoc Imaging System (Bio-Rad) and analyzed using the Image Lab software (Bio-Rad).

### Real-time PCR

Determination of *Sell*, *Klf2*, *Foxo1*, *Bcor*, *S1pr1*, *S1pr3* and *Grk2* expression: RNA was prepared from 1-2x10^6^ magnetically-purified splenic B cells using the Quick-RNA Microprep kit (Zymo Research). cDNA synthesis was performed using random hexamers and the RevertAid First Strand cDNA synthesis Kit (ThermoFisher). Levels of mRNAs were assessed using AceQ SYBR qPCR Master mix (Vazyme) and the primer pairs indicated below on a QuantStudio 7 Flex instrument (ThermoFisher). Samples were run in duplicates and analyzed using the QuantStudio software. Transcript abundance in mutant B cells was normalized to *Hprt* mRNA levels as well as to their expression in control cells. Primers used to detect: *Sell* (5’-CTTACTGGGGCTCGAGGAAC and 5’-TCCAACAGTGAGTTCCATGGT), *Klf2* (5’-CTGCGTACACACACAGGTGAGAA and 5’-AAGTGGCACTGAAAGGGTCTG), *Foxo1* (5’-CGGCGGGCTGGAAGAATT and 5’-TTCTCCGGGGTGATTTTCCG), *Bcor* (5’-AGCCAAAGTCAGTCACCCTG and 5’-TGTCCTCTGGGGCTTCAAAG), *S1pr1* (5’-CGGTGTAGACCCAGAGTCCT and 5’-AGCAGCAGATGAGAATGAAC) (32), *S1pr3* (5’-AGTCTTAGCTGAGACACGGC and 5’-GCGCCAGGAACGTTCATTTC), *Grk2* (5’-TCTGGAGGACCGAGGAGAAG and 5’-AGGCAGAAGTCCCGGAAAAG) and *Hprt* (5’-TCCTCCTCAGACCGCTTTT and 5’-CCTGGTTCATCATCGCTAATC).

Evaluation of miR-142-3p levels: RNA was isolated from FACS-sorted splenic CD19^+^B220^+^CD93^–^IgM^+^CD1d^+^ Fo B cells using the Direct-zol RNA Microprep kit (Zymo Research). miR-142-3p and RNA U6 were specifically reverse transcribed out of 10 ng of total RNA using TaqMan MicroRNA assay (AssayID 000464 and 001973, respectively) and TaqMan MicroRNA reverse Transcription kit (ThermoFisher). Expression of these RNAs was assessed in duplicates using the primers from the TaqMan MicroRNA assay and TaqMan Fast Advanced Master Mix on QuantStudio 7 Flex instrument. Data were analyzed using the QuantStudio software. miR-142-3p abundance was normalized to RNA U6 levels.

### Statistics and graphs

The Prism software (Graphpad) was used to create graphs, calculate means as well as standard deviations, and determine statistical significance using one-way ANOVA with Tukey’s *post hoc* multiple comparisons test, two-tailed unpaired Student’s *t* test and one sample *t* test against a theoretical mean of one.

## Results

### miR-142 supports the generation of a proper mature B compartment

**Figure 1:**
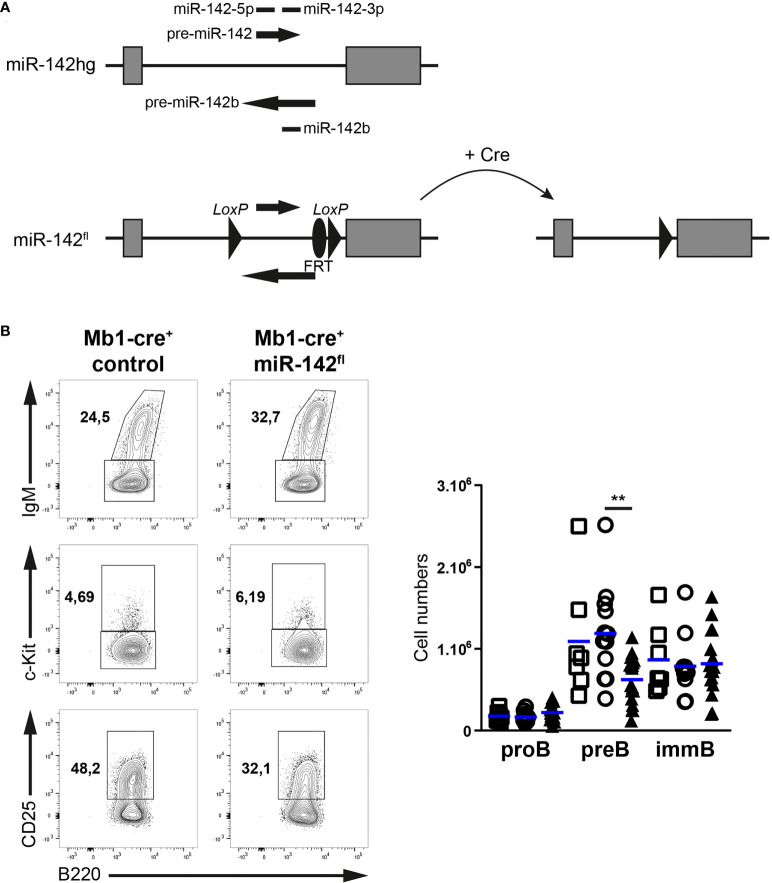
Pre-B cellularity is reduced in Mb1-cre^+^ miR-142^fl^ mice. **(A)** Scheme of the conditional gene targeting strategy: a 677 bp fragment of the miR-142 host gene (miR-142hg) containing the sequences of the precursor and mature forms of miR-142 and miR-142b was flanked with loxP sites (miR-142^fl^) to permit Cre-mediated elimination. FRT site: remnant of a deleted selection cassette used during the work with embryonic stem cells. **(B)** Left: flow cytometry of IgM^+^ immature B cells within CD19^+^B220^+^CD93^+^ progenitor B cells, as well as c-Kit^+^ pro-B cells and CD25^+^c-Kit^–^ pre-B cells within IgM^–^ progenitor B cells in the bone marrow of Mb1-cre^+^ control and Mb1-cre^+^ miR-142^fl^ mice. Right: bone marrow pro-, pre- and immature B cell numbers in Mb1-cre^–^ (□), Mb1-cre^+^ (○) and Mb1-cre^+^ miR-142^fl^ (▲) mice. *n* = 7-18 per group in 18 independent experiments. Each symbol represents one mouse and horizontal blue lines indicate the mean. **, *P* ≤ 0.01 by one-way ANOVA.

While the mature B compartment is altered in miR-142-null mice (17), the individual contributions of the miR-142 locus specifically to B cell physiology remain obscure. In addition, progenitor B cells lacking Dicer demonstrate an enrichment for miR-142-3p targets within upregulated genes (10), suggesting a role for this locus in B cell development. However, the various stages of the latter process have not been examined in the BM of miR-142-null mice. Thus, to gain insights into the cell intrinsic roles of the miR-142 locus in B cells, we generated a mouse allele (miR-142^fl^) permitting the conditional elimination of the pre-miRNAs and mature miR-142-5p, miR-142-3p and miR-142b (**Figure 1A**), and crossed it to the Mb1-cre mouse strain to inactivate this locus specifically in the B lineage (23).

**Figure 2:**
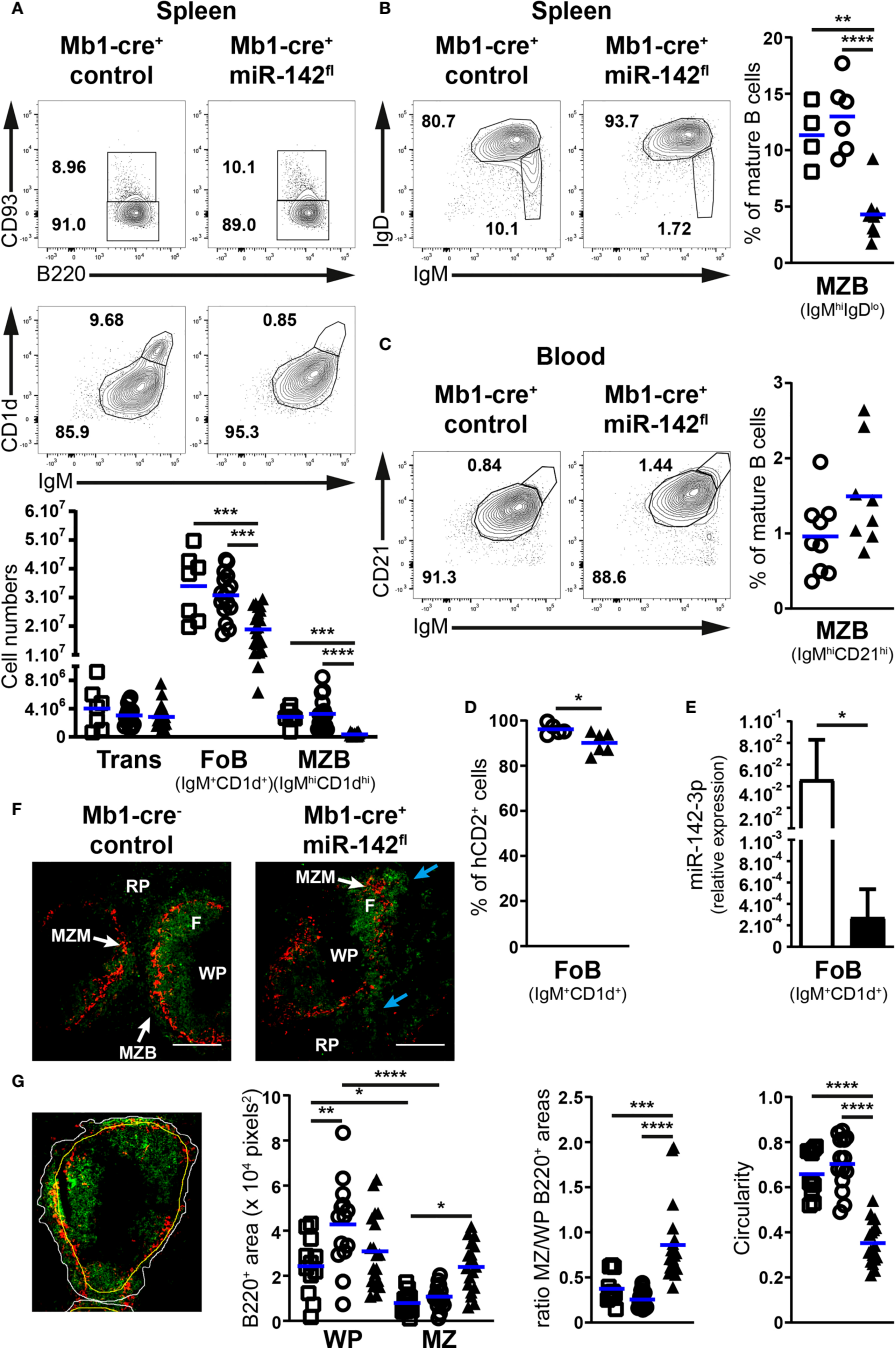
The mature B cell compartment is perturbed in the spleens of Mb1-cre^+^ miR-142^fl^ mice. **(A)** Top: FACS analysis of B220^+^CD93^+^ transitional and B220^+^CD93^–^ mature B cells within splenic CD19^+^B220^+^ B cells, as well as IgM^+^CD1d^+^ follicular and IgM^hi^CD1d^hi^ marginal zone B cells within mature B cells of Mb1-cre^+^ control and Mb1-cre^+^ miR-142^fl^ animals. Bottom: Transitional (Trans), follicular (FoB) and marginal zone (MZB) B cell numbers in the spleens of Mb1-cre^–^ (□), Mb1-cre^+^ (○) and Mb1-cre^+^ miR-142^fl^ (▲) mice. *n* = 7-21 per group in 21 independent experiments. **(B)** Left: flow cytometry of IgM^+^IgD^+^ follicular and IgM^hi^IgD^lo^ marginal zone B cells within splenic CD19^+^B220^+^CD93^–^ mature B cells of control and mutant mice. Right: splenic MZB cell frequency in the mouse groups defined in **(A)**. *n* = 4-8 per group, cumulative of 9 independent experiments. **(C)** Left: FACS analysis of IgM^+^CD21^+^ follicular and IgM^hi^CD21^hi^ marginal zone CD19^+^B220^+^CD93^–^ mature B cells in the blood of control and mutant mice. Right: proportions of MZB cells in the blood of Mb1-cre^+^ (○) and Mb1-cre^+^ miR-142^fl^ (▲) animals. *n* = 8-9 per group, cumulative of 9 independent experiments. **(D)** Proportions of hCD2^+^ Fo B cells in the spleen of Mb1-cre^+^ (○) and Mb1-cre^+^ miR-142^fl^ (▲) mice, as determined by flow cytometry. *n* = 5-6 per group, pooled from 6 independent experiments. **(E)** Real-time PCR of miR-142-3p levels in flow cytometry-isolated splenic IgM^+^CD1d^+^ follicular B cells from Mb1-cre^–^ and Mb1-cre^+^ miR-142^fl^ mice, normalized to the expression of RNA U6. *n* = 4 per group in 3 independent FACS sorts. **(F)** Immunofluorescence confocal microscopy of CD169^+^ (red) macrophages and B220^+^ (green) B cells in spleens from Mb1-cre^–^ control and Mb1-cre^+^ miR-142^fl^ mice. Illustrative of 2 Mb1-cre^–^, 3 Mb1-cre^+^ and 5 Mb1-cre^+^ miR-142^fl^ animals in 4 independent experiments. F, follicle; MZM, marginal zone macrophages; MZB, marginal zone B cells; RP, red pulp; WP, white pulp. Blue arrows, irregular distribution of mutant B cells in the MZ. Magnification, 10x. Scale bar, 200 μm. **(G)** Left: Identification of the perimeter (white) of the B cell population in the marginal zone, of the CD169^+^ macrophage ring (yellow) and of B220 signals (green), as illustrated in an immunofluorescence image of a Mb1-cre^+^ control mouse. Right: B220^+^ areas within the white pulp (WP) and the marginal zone (MZ), ratios of the B220^+^ areas in the MZ and WP, as well as the circularity of the B cell perimeters in 12-19 selected fields of spleen sections from the groups of mice mentioned in **(A)**. **(A–D)** Each symbol depicts one mouse. Means are indicated by horizontal blue lines **(A–D)**, as well as bars plus standard deviations **(E)**. *, *P* ≤ 0.05; **, *P* ≤ 0.01; ***, *P* ≤ 0.001; ****, *P* ≤ 0.0001 by one-way ANOVA **(A, B, G)** and Student’s *t* test **(C–E)**.

**Figure 3:**
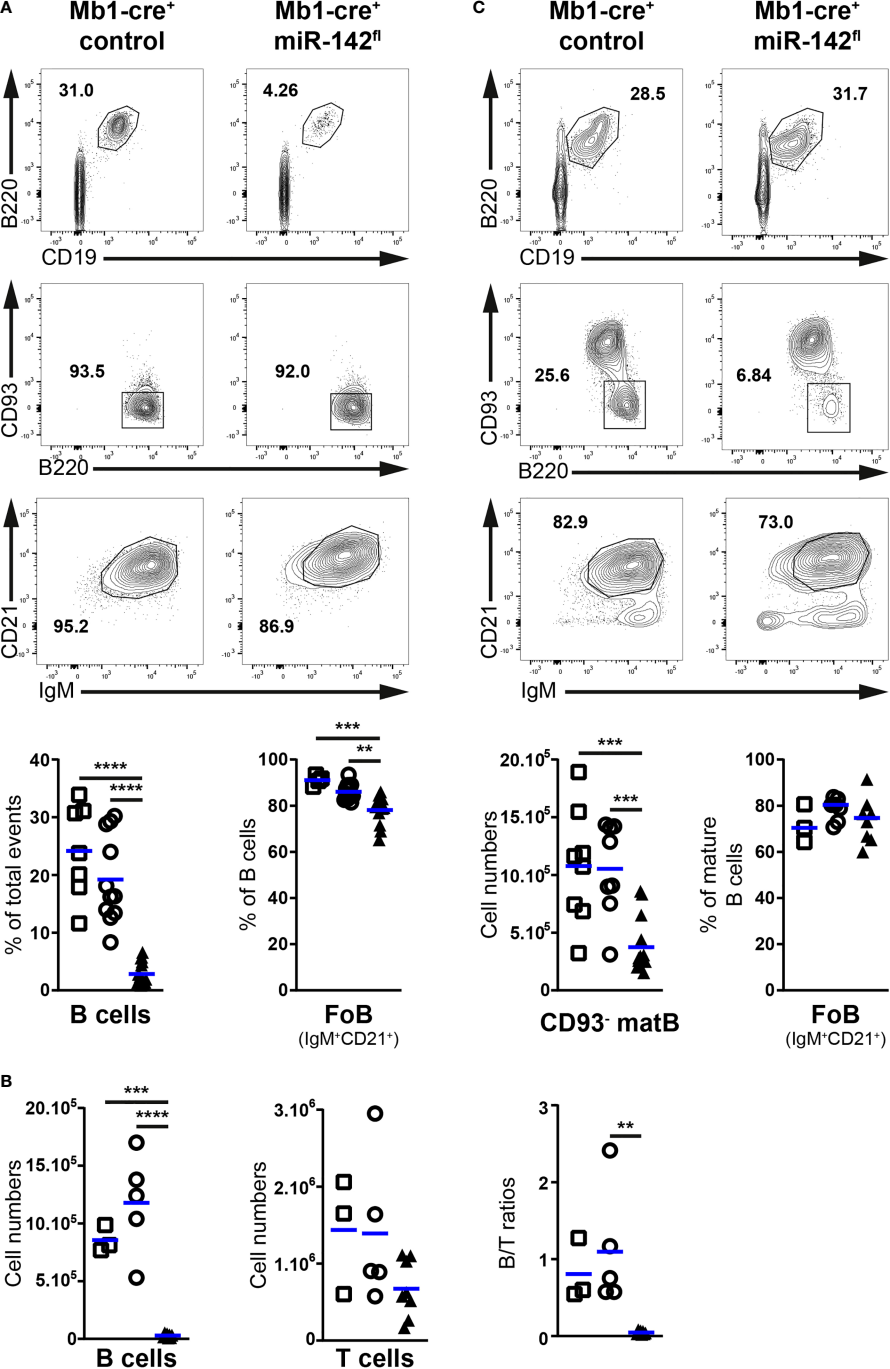
Paucity of follicular B cells in the lymph nodes and bone marrow of Mb1-cre^+^ miR-142^fl^ mice. **(A)** Top: Flow cytometry of IgM^+^CD21^+^ follicular B cells within mature CD19^+^B220^+^CD93^–^ B cells in the lymph nodes (LNs) of Mb1-cre^+^ control and Mb1-cre^+^ miR-142^fl^ mice. Bottom: Proportions of B cells in LNs and follicular (FoB) B cells within LN B cells of Mb1-cre^–^ (□), Mb1-cre^+^ (○) and Mb1-cre^+^ miR-142^fl^ (▲) mice. *n* = 4-16 per group, cumulative of 14-17 independent experiments. **(B)** (Left) CD19^+^ B cell and CD5^+^ T cell numbers, and (right) B/T ratios (% CD19^+^/% CD5^+^ cells within lymphocyte gates), in the inguinal LNs of control and mutant mice, as defined in **(A)**. *n* = 3-8 per group, cumulative of 8 independent experiments. **(C)** Top: FACS analysis of IgM^+^CD21^+^CD19^+^B220^+^CD93^–^ mature follicular B cells in the bone marrow of Mb1-cre^+^ and Mb1-cre^+^ miR-142^fl^ animals. Bottom: mature B cell numbers (CD93^–^ matB) and frequency of follicular B cells within mature B cells in the bone marrow of Mb1-cre^–^ (□), Mb1-cre^+^ (○) and Mb1-cre^+^ miR-142^fl^ (▲) mice. *n* = 3-14 per group, from 9-15 independent experiments. **(A–C)** Each symbol indicates one mouse. Horizontal blue lines signify the means. **, *P* ≤ 0.01; ***, *P* ≤ 0.001; ****, *P* ≤ 0.0001 by one-way ANOVA.

We first investigated the early developmental stages (pro-, pre- and immature B cells) in the BM of our mutant mice (**Figure 1B**). Pre-B cell numbers tended to be lower (1.75 – 1.9-fold) in Mb1-cre^+^ miR-142^fl^ compared to control mice (**Figure 1B**). Intriguingly, the pro- and immature B cell compartments seem unaffected by the absence of miR-142 miRNAs in B cells (**Figure 1B**), suggesting a specific role for these miRNAs in the accumulation of pre-B cells. Next, we examined the splenic B populations in Mb1-cre^+^ miR-142^fl^ mice. We predicted increased numbers of maturing transitional B cells as well as a strong accumulation of MZ B cells in these animals, based on the previous analysis of miR-142-null mice (17). Remarkably, similar numbers of transitional B cells were detected in Mb1-cre^+^ miR-142^fl^ animals and controls (**Figure 2A**). There was also no expansion of MZ B cells in the spleens of the conditional mutant mice. Instead, we could not recognize a distinct IgM^hi^CD1d^hi^ MZ B population in the Mb1-cre^+^ miR-142^fl^ animals (**Figure 2A**). Accordingly, mutant MZ B cell numbers were strongly reduced (8- to 9-fold) (**Figure 2A**). We confirmed these results using an alternative identification strategy. The proportions of IgM^hi^IgD^lo^ MZ B cells were similarly decreased in the spleens of Mb1-cre^+^ miR-142^fl^ mice (**Figure 2B**). Mutant MZ B cells do not seem to be misplaced. We did not observe them to be present in the blood (**Figure 2C**). Likewise, an IgM^hi^CD21^hi^ MZ B population was not visible in the BM or LNs of Mb1-cre^+^ miR-142^fl^ mice, as illustrated in panels A and C of figure 3. We can, however, not exclude that mutant MZ B cells aberrantly move to a tissue not examined in our experiments. In contrast to MZ B cells, mature B cells with a Fo B phenotype (IgM^+^CD1d^+^ and IgM^+^IgD^+^) could be clearly identified in the mutant mice (**Figures 2A, B**). However, this population was about 1,7-fold smaller in the spleens of the mutant animals (**Figure 2A**). A sizable fraction of these splenic mutant IgM^+^CD1d^+^ (Fo) B cells displayed low CD23 expression compared to their control counterparts (36% vs 6.8-10%) (**Supplementary Figure 1A**). The levels of CD23 were also mildly reduced on miR-142-deficient compared to control CD23^+^ Fo B cells (**Supplementary Figure 1A**). In contrast, CD21 levels tended to be slightly elevated on the mutant B cells (**Supplementary Figure 1A**). Our observations are consistent with the reported altered expression of these two markers on splenic mature B cells from miR-142-null mice, which Kramer at al. interpreted as an indication of the increased presence of mutant MZ B cells (17). We decided to consider both CD23^+^ and CD23^lo/–^ mature B cells in Mb1-cre^+^ miR-142^fl^ mice as Fo B cells based on their IgM^+^IgD^+^CD1d^+^ phenotype. The vast majority of mutant and control Fo B cells expressed the Cre-inducible reporter gene hCD2, although the frequency was slightly lower for the miR-142-deficient population (**Figure 2D**). Furthermore, levels of miR-142-3p were substantially reduced (~170-fold) in splenic Fo B cells isolated from Mb1-cre^+^ miR-142^fl^ mice compared to control cells (**Figure 2E**). These findings indicate that the mature B compartment in Mb1-cre^+^ miR-142^fl^ mice is mainly composed of cells that had inactivated the miR-142 locus, i.e., had not escaped Cre-mediated recombination. We also assessed the generation of mature B1 cells. These cells were absent in the peritoneal cavity of Mb1-cre^+^ miR-142^fl^ mice (**Supplementary Figure 2**), in agreement with the results of Kramer et al. in null animals (17).

Splenic MZ and Fo B cells are defined by their anatomical locations as well. Thus, we examined B cell positioning in the spleens of our mice by fluorescence microscopy (**Figure 2F**). The spleen architecture of Mb1-cre^+^ miR-142^fl^ animals appeared grossly similar to controls, showing ring-shaped formations of MZ macrophages and Fo B cell follicles in the white pulp (**Figure 2F**). A typical thin smooth layer of MZ B cells surrounding the ring of macrophages could be observed in the controls (**Figure 2F**). Instead, the distribution of mutant B cells in the marginal zone appeared irregular (**Figure 2F**). To quantify splenic B cell positioning, we estimated the areas covered by these cells in the white pulp and marginal zone (**Figure 2G**). In control animals, splenic B cells occupied expectedly more space in the white pulp than in the marginal zone (**Figure 2G**). On average, the area including control B cells in the MZ was equivalent to 25 – 37% of the surface covered by them in the white pulp (**Figure 2G**). Interestingly, B cells appeared more evenly distributed between these two locations in the spleen of Mb1-cre^+^ miR-142^fl^ mice (**Figure 2G**), with the area holding mutant B cells in the MZ corresponding to 86% of the region populated by the latter cells in the white pulp. In addition, B cells tended to occupy more space in the marginal zone of the mutant compared to control animals (**Figure 2G**). Finally, we determined an index of circularity for the border of the B cell population located in the MZ (**Figure 2G**). A value of 1 represents a perfect (smooth) circle. This index was noticeably lower in the mutant animals, in agreement with a more irregular distribution of B cells in the region of the marginal zone. Thus, these data are consistent with the flow cytometry analysis suggesting the absence of a bona fide MZ B population and the presence of Fo B cells in Mb1-cre^+^ miR-142^fl^ mice.

Since several of our observations on B cells in Mb1-cre^+^ miR-142^fl^ mice differ from published work in miR-142-null mice (17, 19), we evaluated whether the discrepancies were caused by the design of our mutant allele. Thus, we crossed our miR-142^fl^ mice with a deleter-cre strain to generate animals deficient for the miR-142 locus in the germline (22). Consistent with the literature, we observed an accumulation of splenic B cells in our miR-142^–/–^ mice (**Supplementary Figure 3A**). However, this higher cellularity appeared to result from the expansion of mature IgM^+^CD1d^+^ (Fo) B cells in our mice (**Supplementary Figure 3A**), instead of the anticipated MZ B cells (17). We could not detect a distinct IgM^hi^CD1d^hi^ MZ B cell population in our miR-142-null animals (**Supplementary Figure 3A**), similar to the situation in Mb1-cre^+^ miR-142^fl^ mice (**Figure 2**). Since no information is available, we also examined the early stages of B cell development in the BM of our miR-142 knockout mice. These mice demonstrated higher immature B cell numbers than controls (means of 2.1x10^6^ vs 1.2x10^6^), while pro- and pre-B cellularity appeared normal (**Supplementary Figure 3B**). Unexpectedly, we detected a clear pre-B cell population in the spleens of our miR-142^–/–^ animals (**Supplementary Figure 3B**). The results are compatible with enhanced production of B cells in miR-142-null mice, which could participate in the increased pool of splenic mature mutant B cells alongside the proposed pro-survival contribution of higher BAFFR levels (17). Of note, T cell numbers were reduced and neutrophil cellularity was increased in the spleens of our miR-142^–/–^ mice (**Supplementary Figures 3C, D**), consistent with previous work (16, 17, 19). Thus, the phenotype of our miR-142-deficient mice resembles the one reported in other null mice (16, 17, 19), excluding potential flaws in the design of the miR-142^fl^ allele and validating our findings in Mb1-cre^+^ miR-142^fl^ animals.

Overall, our results indicate cell autonomous roles for the miR-142 locus at several stages of B cell development. Thus, the inactivation of this locus selectively in B cells led to an undersized pre-B cell population and a lack of B1 cells. In addition, an aberrant Fo B population was detected in Mb1-cre^+^ miR-142^fl^ mice, with reduced cellularity and abnormal expression of CD23. Also, genuine MZ B cells could not be observed by flow cytometry or immunofluorescence microscopy in the mutant animals.

### Paucity of mature B cells in the bone marrow and lymph nodes of Mb1-cre^+^ miR-142^fl^ mice

Since Fo B cells circulate between lymphoid tissues (1, 13), we examined the size of the mature population in peripheral LNs and BM upon conditional ablation of the miR-142 locus in B cells. Remarkably, the frequency of LN B cells was strongly decreased in Mb1-cre^+^ miR-142^fl^ compared to control mice (6,8- to 8,5-fold on average) (**Figure 3A**). Fo B cells, detected as IgM^+^CD21^+^ mature B cells, were the dominant B cell population in the LNs of all groups of mice (**Figure 3A**), implying a specific loss of LN Fo B cells in the mutant animals. In agreement, evaluations of CD19^+^ B cellularity in the inguinal LNs indicated that these tissues contained only 2.8x10^4^ B cells in Mb1-cre^+^ miR-142^fl^ mice compared to 8.5-11.8x10^5^ in controls (**Figure 3B**). Inguinal LN CD5^+^ T cellularity was not significantly impacted in the mutant mice (**Figure 3B**). Consistently, the B to T cell ratios in these tissues dropped substantially in the Mb1-cre^+^ miR-142^fl^ mice (**Figure 3B**). The limited impact on T cell numbers suggests that the paucity of B cells is not an indirect result of dysfunctional LNs in the mutant mice (**Figure 3B**). Similar to the spleen, a substantial fraction of LN Fo B cells was CD23^lo/–^ in Mb1-cre^+^ miR-142^fl^ mice (**Supplementary Figure 1B**). However, surface levels of CD23 and CD21 were not noticeably altered on LN mutant CD23^+^ and total Fo B cells respectively (**Supplementary Figure 1B**), differing from the observations made on their splenic counterparts (**Supplementary Figure 1A**). Mb1-cre^+^ miR-142^fl^ mice also demonstrated a reduction (3-fold) of mature Fo B cellularity in the BM (**Figure 3C**). Proportions of CD23^lo/–^ Fo B cells tended to be higher in the BM of the mutant mice as well, although the increase did not reach significance (**Supplementary Figure 1C**).

Thus, the overall numbers of Fo B cells appear more affected in the BM and especially LNs than in the spleens of Mb1-cre^+^ miR-142^fl^ mice. These findings point to a possible role for the miR-142 locus in the regulation of naïve Fo B cell migratory properties.

### Mutant follicular B cells respond efficiently to lymph node homing chemokines

The miR-142 locus has been invoked in the modulation of mouse neutrophil and CD4^+^ T cell migration (33, 34). Focusing on the LNs, we hypothesize that altered responses to chemokines could contribute to the greatly reduced numbers of mutant B cells in these tissues.

**Figure 4:**
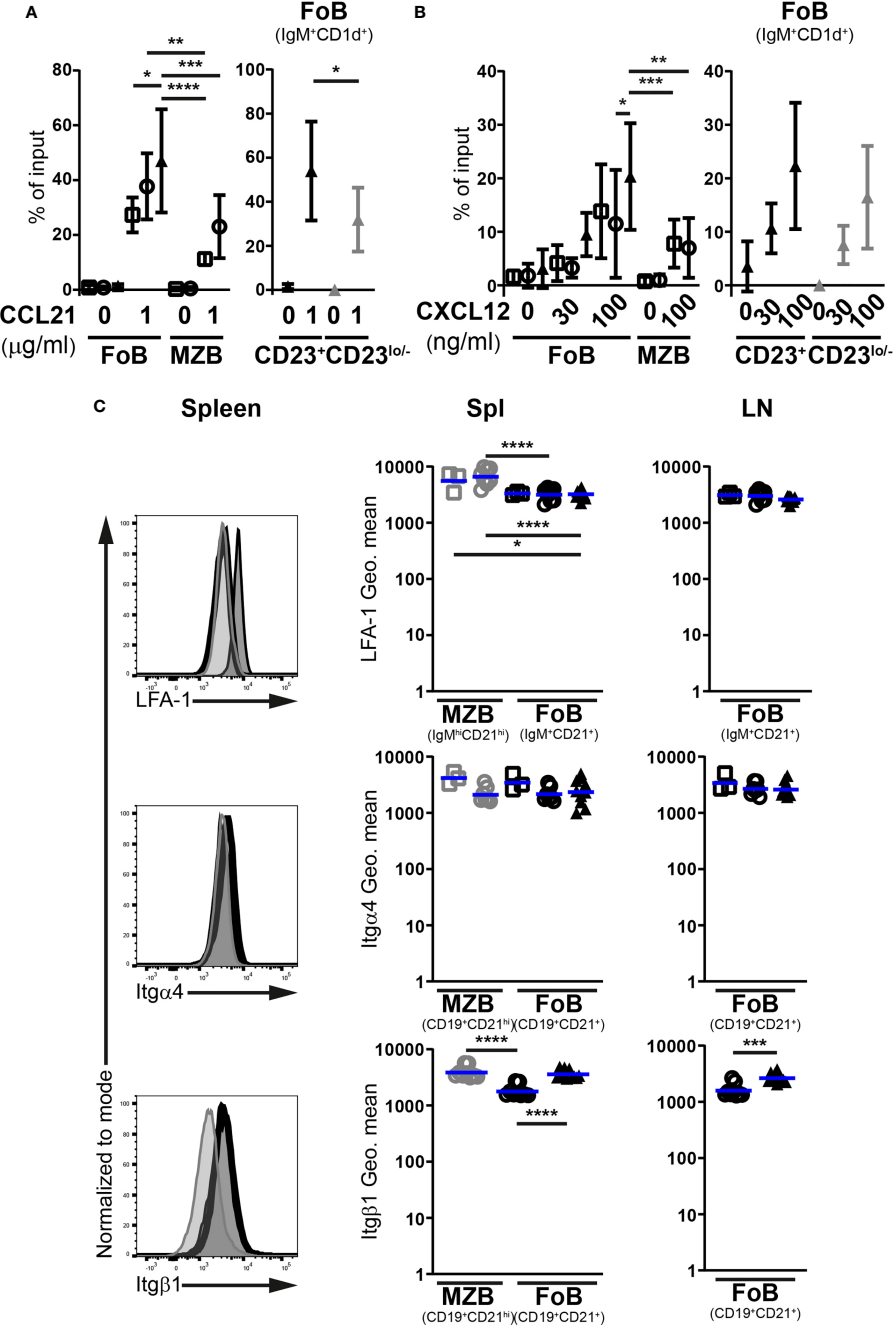
miR-142-deficient follicular B cells do not show signs of compromised ability to firmly adhere to high endothelial venules. *In vitro* migration of splenic IgM^+^CD1d^+^ follicular (FoB) and IgM^hi^CD1d^hi^ marginal zone (MZB) CD19^+^B220^+^CD93^–^ mature B cells, as well as CD23^+^ and CD23^lo/–^ mutant Fo B cells from Mb1-cre^–^ (□), Mb1-cre^+^ (○) and Mb1-cre^+^ miR-142^fl^ (▲) mice in response to **(A)** 1 μg/ml CCL21 (*n* = 4-7 per group in 9 independent experiments) as well as **(B)** 30 and 100 ng/ml CXCL12 (*n* = 5-12 per group, cumulative of 14 independent experiments). B cells were identified by flow cytometry. Cells that migrated to the bottom chamber of the transwell after 3h are given as proportions of the specific B population in the input splenocytes (% of input). **(C)** Left: flow cytometry of the expression of LFA-1, integrin α4 (Itgα4) and integrin β1 (Itgβ1) at the surface of splenic IgM^+^CD21^+^ or CD19^+^CD21^+^ Fo B cells (thick black histogram) from Mb1-cre^+^ miR-142^fl^ mice, as well as on splenic IgM^+^CD21^+^ or CD19^+^CD21^+^ Fo (light grey-filled histogram) and IgM^hi^CD21^hi^ or CD19^+^CD21^hi^ MZ (dark grey-filled histogram) B cells from Mb1-cre^+^ control animals. Right: Geometric means (Geo. mean) of surface LFA-1, Itgα4 and Itgβ1 on Fo (black) and MZ (grey) B cells from spleens (Spl) and lymph nodes (LN) of Mb1-cre^–^ (□), Mb1-cre^+^ (○) and Mb1-cre^+^ miR-142^fl^ (▲) mice. *n* = 3-11 per group in 6-7 independent experiments. **(A, B)** Symbols and vertical lines indicate the means and standard deviations respectively. **(C)** Each symbol represents one mouse. Horizontal blue lines signify the means. *, *P* ≤ 0.05; **, *P* ≤ 0.01; ***, *P* ≤ 0.001; ****, *P* ≤ 0.0001 by one-way ANOVA **(A–C)** or Student’s *t* test **(C)**. **(A, B)** Data on Fo B cell migration in absence of treatment from 4 Mb1-cre^–^, 6 Mb1-cre^+^ and 7 Mb1-cre^+^ miR-142^fl^ mice are shared between the 2 graphs. **(B)** Samples excluded from the analysis: One mutant Fo B cells (100 ng/ml CXCL12) for an aberrant high value. One Mb1-cre^–^ and 3 Mb1-cre^+^ MZ B cells (100 ng/ml CXCL12) for insufficient events in the gate.

Both CCR7 and CXCR4 promote the homing of naïve Fo B cells to LNs, with signals from CCR7 seemingly playing a more important role (35, 36). Accordingly, we determined the chemotactic responses of splenic IgM^+^CD1d^+^ Fo B cells towards CCL21 and CXCL12, performing *in vitro* transwell assays (**Supplementary Figure 4**). Control IgM^hi^CD1d^hi^ MZ B cells were included in these analyses for comparison since these cells exhibit migratory properties distinct from Fo B cells. In the absence of chemokines, the random access of mutant Fo B cells to the bottom chamber of the transwell was minimal and comparable to control Fo B cells (**Figures 4A, B**). miR-142-proficient and -deficient Fo B cells migrated similarly in the presence of CCL21 (1 μg/ml), although the chemotactic response of the mutant cells appeared slightly better (**Figure 4A**). Both types of Fo B cells demonstrated higher sensitivity to CCL21 signals than control MZ B cells (**Figure 4A**). These results are consistent with the reported chemotactic responses of wild-type splenic mature B populations to CCL19, a second CCR7 ligand (37, 38). In addition, we tested the possibility of distinct behaviors for mutant Fo B cells expressing high and low levels of CD23 in CCL21-mediated chemotaxis. CD23^lo/–^ miR-142-deficient Fo B cells migrated slightly less than their CD23^+^ counterparts in presence of the chemokine (**Figure 4A**). Yet, proportions of migrating mutant CD23^lo/–^ B cells remained within the range seen for control Fo B cells, excluding a compromised response to CCL21.

Mature B cell responses to CXCL12-mediated chemotaxis were also consistent with previous work (**Figure 4B**) (29). Both control Fo and MZ B cells migrated towards CXCL12 (100 ng/ml), with the former cells tending to be better responders (**Figure 4B**). The mutant Fo B cells seemed to migrate on average slightly more than their control counterparts (**Figure 4B**). Similar observations were made at a lower CXCL12 concentration (30 ng/ml) (**Figure 4B**). miR-142-deficient Fo B cells displayed some mild chemotactic response, while control cells barely migrated. Within the miR-142-deficient Fo B population, the CD23^+^ and CD23^lo/–^ cells did not obviously differ in their sensitivity to CXCL12 signals (**Figure 4B**), although the mutant CD23^lo/–^ B cells showed a modest tendency to migrate less well in presence of the chemokine.

Since the above *in vitro* migration assays suggest functional CCR7 and CXCR4 signaling, the expression of the LFA-1 (αLβ2) integrin could instead be affected upon inactivation of the miR-142 locus in B cells. A minor participation for α4-containing integrin, like VLA-4 (α4β1), interaction with VCAM1 in the firm adhesion of lymphocytes to HEVs has also been reported (39). We therefore monitored surface levels of LFA-1, Itgα4 and Itgβ1 on Fo B cells (**Figure 4C**). As expected (40), the abundance of LFA-1 and Itgβ1 was higher on MZ compared to Fo B cells, while Itgα4 levels were similar, in the spleens of control mice. The surface expression of these molecules was, however, not lower on the mutant Fo B cells compared to their control counterparts in either the spleen or LNs. Itgβ1 expression was even higher (1.5 – 2-fold) on the mutant cells, reaching levels observed for control MZ B cells (**Figure 4C**).

Overall, the responsiveness of splenic Fo B cells from Mb1-cre^+^ miR-142^fl^ mice to chemotactic signals from both CCL21 and CXCL12 *in vitro* appears comparable to their control counterparts. The levels of integrins mediating the attachment of B cells to the HEVs were also not decreased on the mutant cells. Thus, defective firm adhesion during homing seems unlikely the cause of the LN B cell paucity in the mutant animals.

### Impaired CD62L expression on follicular B cells from Mb1-cre^+^ miR-142^fl^ mice

Since the firm adhesion to HEVs of mutant Fo B cells does not appear problematic, the rolling/tethering step during their homing to LNs could be compromised instead. This latter process depends on the expression of CD62L by immune cells (5, 41, 42). Indeed, CD62L-null mice demonstrate a drastic reduction in peripheral LN cell content (43).

**Figure 5:**
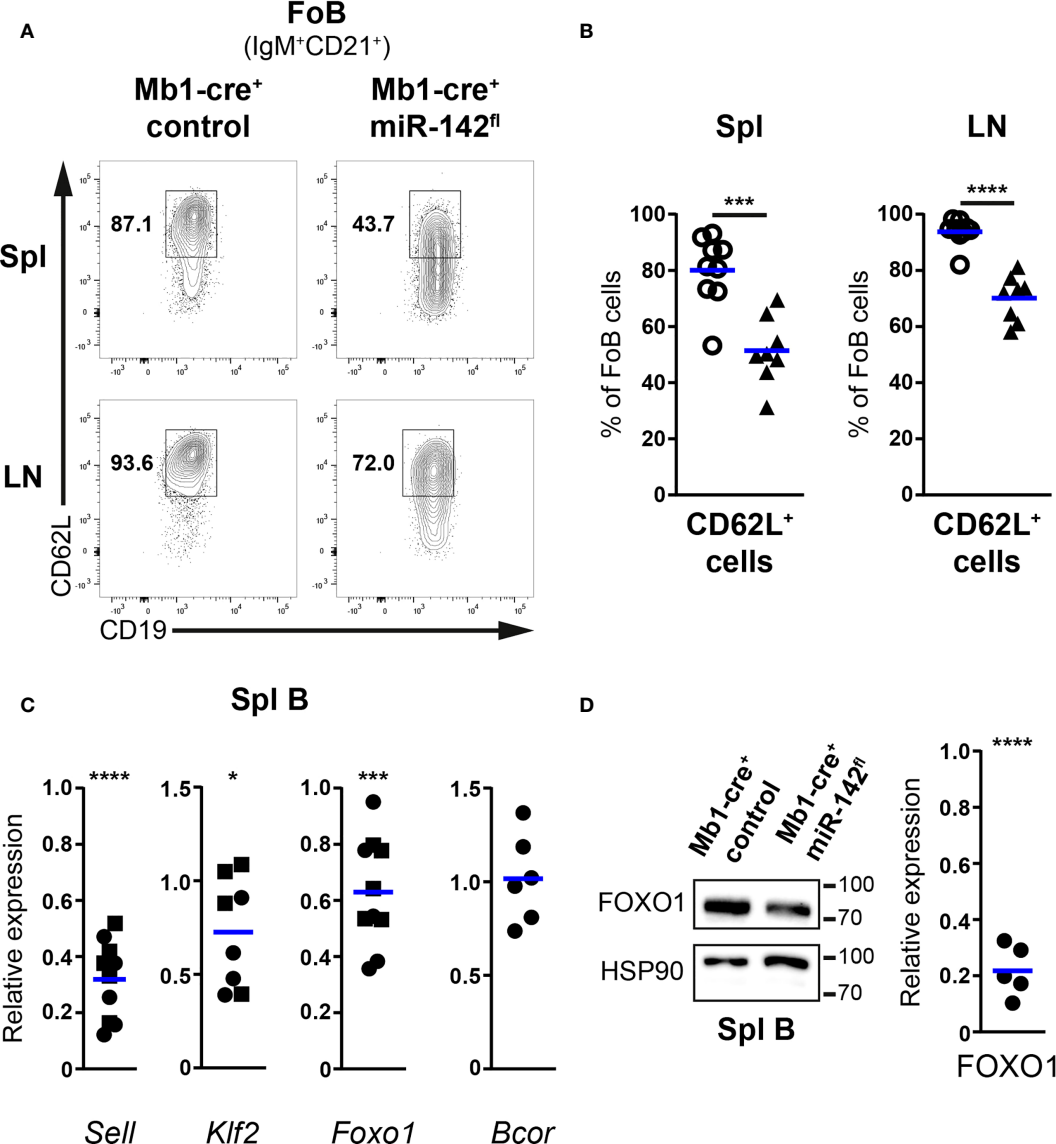
CD62L expression is impaired in follicular B cells of Mb1-cre^+^ miR-142^fl^ mice. **(A)** flow cytometry of CD62L^+^ cells within IgM^+^CD21^+^CD19^+^B220^+^CD93^–^ follicular B (FoB) cells in the spleens (Spl) and lymph nodes (LN) of Mb1-cre^+^ control and Mb1-cre^+^ miR-142^fl^ mice. **(B)** Proportions of CD62L^+^ Fo B cells in Spl and LN of Mb1-cre^+^ (○) and Mb1-cre^+^ miR-142^fl^ (▲) animals. *n* = 8-9 per group in 9 independent experiments. **(C)** Real-time PCR of *Sell*, *Klf2*, *Foxo1* and *Bcor* mRNA levels in magnetically-purified splenic B cells from Mb1-cre^+^ miR-142^fl^ mice. Normalized to *Hprt* transcript levels and to their respective expression in Mb1-cre^–^ (*n=4-5*, ◼) and Mb1-cre^+^ (*n=4-6*, ●) controls. Cumulative of 15 independent pairs of mice. **(D)** Left: Western blotting of FOXO1 and loading control HSP90 in splenic B cells magnetically-isolated from Mb1-cre^+^ and Mb1-cre^+^ miR-142^fl^ mice. Right: Quantification of FOXO1 protein levels in splenic B cells from Mb1-cre^+^ miR-142^fl^ animals. Normalized to the expression of HSP90 and to the relative levels of FOXO1 in Mb1-cre^+^ controls. *n* = 5, pooled from 5 independent pairs of mice. Each symbol represents one mouse **(B)** or a ratio between a pair of control and mutant animals **(C, D)**. **(B–D)** Horizontal blue lines indicate means. *, *P* ≤ 0.05; ***, *P* ≤ 0.001; ****, *P* ≤ 0.0001 by Student’s *t* test **(B)** and one sample *t* test **(C, D)**. **(C)** Excluded from the analyses: two *Klf2* datapoints due to bad signals in the control samples.

Proportions of Fo B cells positive for CD62L were on average reduced by 30% in the spleens of Mb1-cre^+^ miR-142^fl^ mice compared to controls (51,4% vs 80.04%) (**Figures 5A, B**). Similar observations were made in the LNs (**Figures 5A, B**). Of note, the fraction of CD62L^+^ mutant Fo B cells was higher in the LNs than in the spleens, consistent with a need for lymphocytes to express sufficient L-selectin to enter the former tissues. In comparison to controls, surface levels of L-selectin also tended to be lower (1.5- to 2-fold) on CD62L^+^ mutant Fo B cells, in particular in the LNs (**Supplementary Figure 5A**). Next, we evaluated whether cells with decreased surface L-selectin would be mainly found within CD23^lo/–^ mutant Fo B cells. Unexpectedly, splenic and LN proportions of CD62L^+^ cells were diminished in CD23^lo/–^ compared to CD23^+^ Fo B populations for both control and mutant mice (**Supplementary Figure 5B**). Nevertheless, frequencies of CD62L^+^ cells were consistently lower for the mutant B cells independently of their levels of CD23 (**Supplementary Figure 5B**). Thus, the reduction in L-selectin affects the whole mutant Fo B population and does not correlate with changes in CD23 expression.

We next attempted to identify molecular alterations that could lead to the impaired display of surface CD62L on mutant B cells. Levels of the CD62L transcript (*Sell*) in splenic B cells from Mb1-cre^+^ miR-142^fl^ mice were only about 32% of the expression observed in controls by real time PCR (**Figure 5C**). These results are compatible with inefficient transcription of L-selectin in the mutant Fo B cells. FOXO1 and KLF2 promote the transcription of *Sell* in mouse B cells (44–47). FOXO1 can also modulate to some extent *Klf2* expression (45). Thus, we examined the levels of these two transcription factors in the B cells from our mutant mice. *Klf2* and *Foxo1* mRNA levels were moderately decreased by on average 30% and 40%, respectively, in splenic miR-142-deficient compared to proficient B cells (**Figure 5C**). The polycomb repressive complex 1.1 has been reported to participate in establishing a closed chromatin conformation at the Foxo1 locus in acute myeloid leukemia blasts (48). A component of the latter complex is BCOR, a predicted miR-142-5p target (Targetscan.org). Increased BCOR amount could thus contribute to limit *Foxo1* expression in our mutant B cells due to enhanced formation of polycomb complexes. A major effect of eliminating miRNA-mediated repression is the improved stability of target transcripts (9). Yet, no clear increase in the abundance of *Bcor* was detected in splenic mutant B cells (**Figure 5C**), suggesting that this repressor may not be regulated by miR-142-5p in mature B cells. In agreement with the above results, the amount of FOXO1 was also reduced in splenic miR-142-deficient B cells compared to controls, by Western blotting (**Figure 5D** and **Supplementary Figure 6**). The relative reduction in FOXO1 protein appeared however much stronger than the decrease seen at the transcript levels in the mutant B cells, implying a possible additional defective regulation of FOXO1 stability in the latter cells.

In summary, the miR-142 locus appears involved in establishing the typical surface levels of CD62L seen on Fo B cells. In particular, these miRNAs seem to favor the production of FOXO1, ultimately permitting an optimal transcription of *Sell*. These findings also support a contribution for impaired B cell tethering/rolling in HEVs to the reduced size of the mutant LN B population.

### Compromised accumulation of mutant B cells in a normal lymph node environment

**Figure 6:**
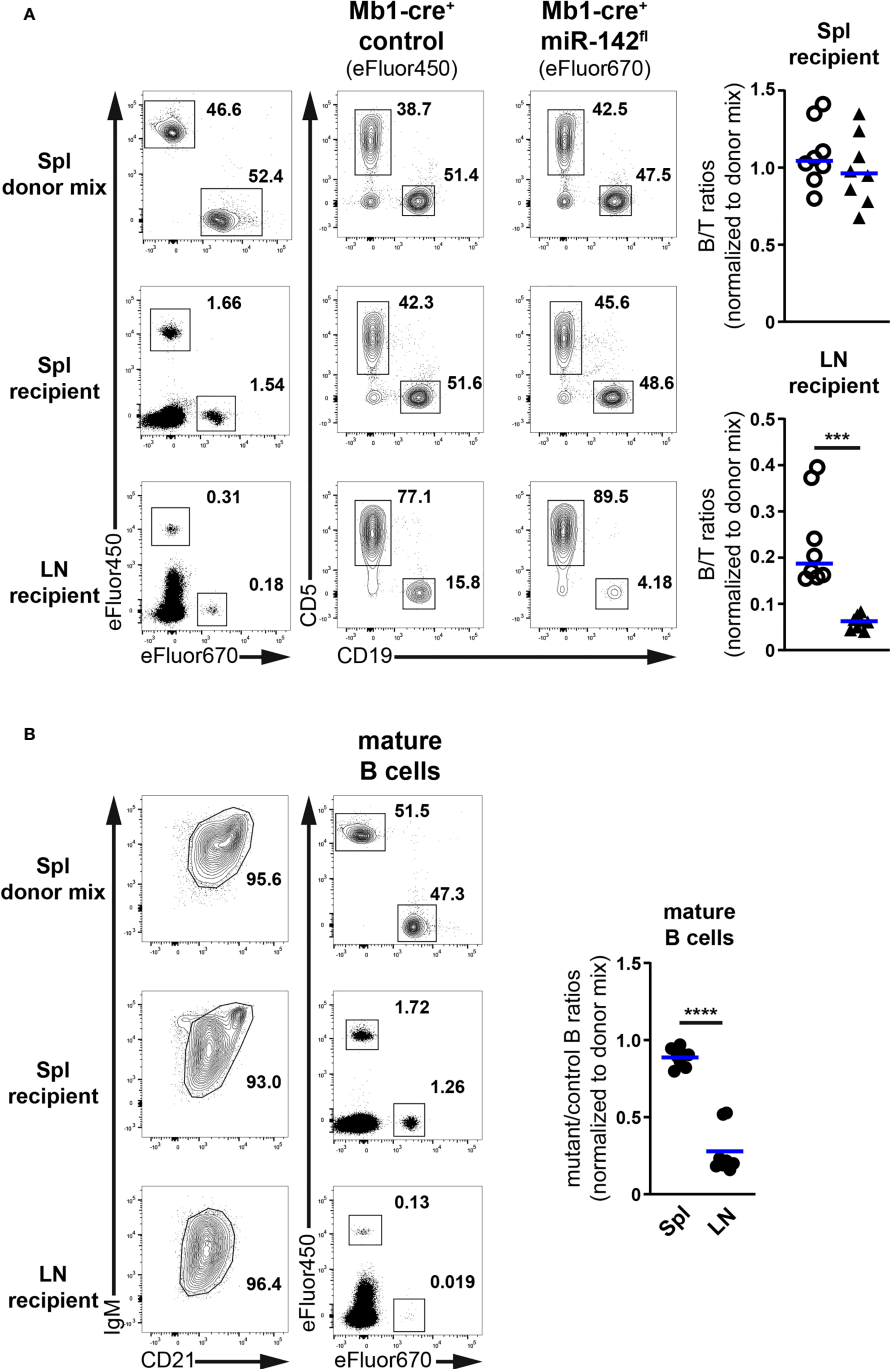
Limited accumulation of miR-142-deficient B cells in normal peripheral lymph nodes. **(A)** Left: flow cytometry of CD5^+^ T and CD19^+^ B cells within Cell proliferation Dye eFluor450 (Mb1-cre^+^ control) and eFluor670 (Mb1-cre^+^ miR-142^fl^) labelled cells in the splenocyte (Spl) donor mixes, as well as in the spleens (Spl) and lymph nodes (LN) of the recipient animals after adoptive transfer. Right: B/T ratios within cells from Mb1-cre^+^ control (○) and Mb1-cre^+^ miR-142^fl^ (▲) mice present in the spleens and lymph nodes of the recipient mice. Normalized to B/T ratios of the donor mixes. **(B)** Left: FACS analysis of labelled Mb1-cre^+^ control (eFluor450) and mutant (eFluor670) within CD19^+^B220^+^CD93^–^IgM^+/hi^CD21^+/hi^ mature B cells in the splenocyte donor mixes, as well as in the spleens and lymph nodes of the recipient animals. Right: mutant/control ratios within mature B cells in the spleens and lymph nodes of the recipients. Normalized to mutant/control mature B cell ratios of the corresponding donor mixes. **(A, B)** Cumulative of 4 independent donor mixes (Mb1-cre^+^ control/Mb1-cre^+^ miR-142^fl^) injected in 2 recipient mice each and performed in two separate experiments. Each symbol indicates one recipient mouse. Horizontal blue lines signify the means. ***, *P* ≤ 0.001; ****, *P* ≤ 0.0001 by Student’s *t* test.

We decided to verify that the migration of miR-142-deficient B cells to the LNs is indeed compromised (**Figure 6**). Splenocytes from control and mutant mice were isolated, labelled with proliferation dyes, mixed in equal parts, and injected into recipient animals. 2h post-transfer, the presence of labelled B and T cells in the spleens and LNs of the recipients was evaluated by flow cytometry. T cells were used as a reference since their physiology is not affected by our targeting strategy.

Proportions of labelled B and T cells in the spleens of recipients were comparable to their frequencies in the donor mixes (**Figure 6A**). Accordingly, the normalized B/T ratios within control and mutant donor cells averaged 1, indicating that these various lymphocyte populations similarly accumulated in the spleen. In the recipient lymph nodes, percentages of labelled T cells were higher while the fractions of labelled B cells were reduced compared to the donor mixes (**Figure 6A**), in agreement with the known propensity of the former cells to efficiently accumulate in LNs. Notably, the proportions of mutant B cells were clearly smaller than their control counterparts, leading to significantly lower (3.9-fold) B/T ratios in the case of mutant compared to control donor cells (**Figure 6A**). These observations reveal an impaired accumulation of miR-142-deficient B cells in the recipient LNs. They also remind one the decreased B/T ratios seen in the inguinal LNs of Mb1-cre^+^ miR-142^fl^ compared to control animals (**Figure 3B**). Likewise, the normalized ratios of labelled mutant to control donor cells within the IgM^+/hi^CD21^+/hi^ mature B populations in the recipients were close to 1 in the context of the spleen whereas the values strongly dropped for the LNs (**Figure 6B**).

Thus, miR-142-deficient mature B cells cannot properly accumulate in LNs. Since the evaluations were done 2h after adoptive transfer, the reduced mutant population size appears primarily the result of defective homing likely due to impaired CD62L expression. In contrast, mutant B cell migration to the spleen seems unperturbed, consistent with L-selectin not being necessary for the entry of lymphocytes into this organ (43).

### miR-142-deficient follicular B cells show increased susceptibility to egress signals

**Figure 7:**
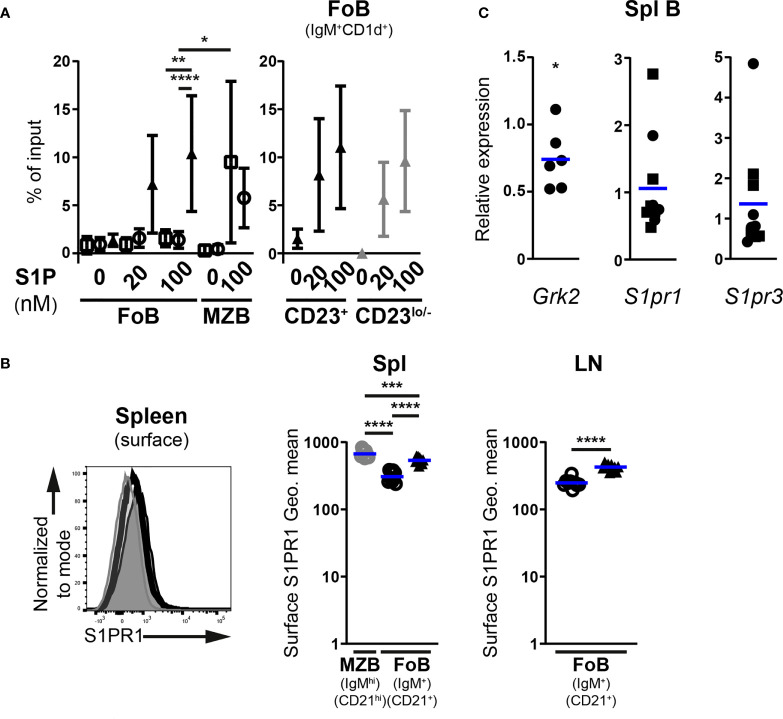
Enhanced S1P-mediated chemotaxis of follicular B cells upon inactivation of the miR-142 locus. **(A)** *In vitro* migration of splenic IgM^+^CD1d^+^ follicular (FoB) and IgM^hi^CD1d^hi^ marginal zone (MZB) CD19^+^B220^+^CD93^–^ mature B cells, as well as CD23^+^ and CD23^lo/–^ mutant Fo B cells from Mb1-cre^–^ (□), Mb1-cre^+^ (○) and Mb1-cre^+^ miR-142^fl^ (▲) mice in response to 20 and 100 nM sphingosine-1-phosphate (S1P) (*n* = 3-9 per group, cumulative of 11 independent experiments). B cells were detected in flow cytometry. Cells that migrated to the bottom chamber of the transwell after 3h are given as proportions of the specific B population in the input splenocytes (% of input). **(B)** Left: Flow cytometry of S1PR1 surface levels on splenic IgM^+^CD21^+^ Fo B cells (thick black histogram) from Mb1-cre^+^ miR-142^fl^ mice, as well as on splenic IgM^+^CD21^+^ Fo (light grey-filled histogram) and IgM^hi^CD21^hi^ MZ (dark grey-filled histogram) B cells from Mb1-cre^+^ control animals. Right: Geometric means (Geo. Mean) of surface S1PR1 on Fo (black) and MZ (grey) B cells from spleens (Spl) and lymph nodes (LN) of Mb1-cre^+^ (○) and Mb1-cre^+^ miR-142^fl^ (▲) mice. *n* = 9-10 per group in 6 independent experiments. **(C)** Real-time PCR of *S1pr1*, *S1pr3* and *Grk2* mRNA levels in magnetically-purified splenic B cells from Mb1-cre^+^ miR-142^fl^ mice. Normalized to *Hprt* transcript levels and to their respective expression in Mb1-cre^–^ (*n=5*, ◼) and Mb1-cre^+^ (*n=5-6*, ●) controls. Pooled from 15 independent pairs of mice. **(A)** Symbols and vertical lines indicate the means and standard deviations respectively. **(B, C)** Horizontal blue lines signify the means. Each symbol represents one mouse **(B)** or a ratio between a pair of control and mutant animals **(C)**. *, *P* ≤ 0.05; **, *P* ≤ 0.01; ***, *P* ≤ 0.001; ****, *P* ≤ 0.0001 by one-way ANOVA **(A, B)**, Student’s *t* test **(B)** and one sample *t* test **(C)**. **(A)** Data on Fo B cell migration in the absence of treatment from 4 Mb1-cre^–^, 6 Mb1-cre^+^ and 7 Mb1-cre^+^ miR-142^fl^ mice are shared with the experiments in **Figure 4**. Samples excluded: One Mb1-cre^+^ Fo B cells (both 20 and 100 nM S1P) as well as one Mb1-cre^–^ and 3 Mb1-cre^+^ MZ B cells (100 nM S1P) for insufficient events within the gate.

Finally, we checked whether the miR-142 locus could also play a role in the regulation of B cell egress from lymphoid tissues. Therefore, we determined the effects of S1P chemotactic signals on Fo B cells from Mb1-cre^+^ miR-142^fl^ mice in transwell assays *in vitro*. Migration of MZ B cells from the spleen of control mice was visibly induced by 100 nM S1P (13-31-fold increased vs no S1P) while the Fo B cells barely responded (**Figure 7A**), as expected from the literature (28, 37). miR-142-deficient Fo B cells migrated clearly more to 100 nM of S1P than their control counterparts and in proportions comparable to MZ B cells (**Figure 7A**). This trend was already observed at a lower concentration (20 nM) of the phospholipid (**Figure 7A**). The improved chemotactic response to S1P did not appear dependent on CD23 expression by the mutant Fo B cells, since both CD23^+^ and CD23^lo/–^ populations reacted similarly to the chemoattractant (**Figure 7A**).

Two receptors for S1P have well known roles in B cell migration. The predominant S1PR1 chiefly regulates the egress of mature B cells from lymphoid tissues *in vivo*, while the lesser expressed S1PR3 mediates S1P chemotaxis *in vitro* (6, 7, 28, 37, 49). The availability of a suitable commercial antibody allowed us to assess S1PR1 abundance on mature B cells by flow cytometry, although the detected signals were dim like in previous work (50, 51). Surface expression of S1PR1 appeared higher (1.7-fold) on mutant Fo B cells compared to controls in the spleen and LNs (**Figure 7B**). This expression did however not reach the levels seen on control MZ B cells (**Figure 7B**), the strongest expressors of S1PR1 among splenic B cells (51).

Absence of the kinase GRK2 in B cells results in higher surface levels of S1PR1 and limits their accumulation in peripheral LNs (50, 51). The kinase also interacts with S1PR3 in endothelial cells (52), suggesting a role for GRK2 in the internalization of the latter receptor as well. We thus examined *Grk2* expression in control and mutant splenic B cells. Transcript levels of the kinase were only modestly reduced (25% on average) in B cells lacking miR-142 miRNAs (**Figure 7C**). It seems unlikely that such a subtle change in expression is the main driver of elevated S1PR1 surface levels on mutant Fo B cells. Therefore, we determined whether the abundance of *S1pr1*, as well as *S1pr3*, may be enhanced in miR-142-deficient cells. Remarkably, no clear accumulation of either receptor’s mRNAs could be observed in splenic mutant B cells compared to controls (**Figure 7C**). Thus, the higher surface levels of S1PR1 on mutant Fo B cells does not appear to stem from the increased transcription of its gene or, since *S1pr1* is a predicted target of miR-142-5p (Targetscan.org, Pictar.mdc-berlin.de), from the relief of miRNA-mediated transcript destabilization. While the role of S1PR3, a possible target of miR-142-3p, in mutant B cell response to S1P is unclear, our data rule out increased mRNA levels as a possible contribution to the perturbed physiology of mature miR-142-deficient B cells.

Collectively, the above findings indicate that the miR-142 locus participates in weakening Fo B cell responsiveness to S1P signals and may thus slow down their exit from LNs *in vivo*. Although the molecular mechanism remains to be uncovered, miR-142 miRNAs could regulate B cell egress *in vivo* by limiting the amount of S1PR1 available at the cell surface.

## Discussion

Our analyses of naive Mb1-cre^+^ miR-142^fl^ mice indicated the contribution of the miR-142 locus to several steps during B cell development. In the BM, we uncovered an unappreciated role for this locus in the modulation of pre-B cellularity (**Figure 1**). This effect appeared to be masked in the miR-142-null mice by a generally increased B cell production leading to an accumulation of pre- and immature progenitor B cells (**Supplementary Figure 3**). The precise process(es) targeted by the miR-142 miRNAs remain(s) to be defined. Nevertheless, immortalized Dicer-deficient pro-B cells demonstrated an enriched signature for miR-142-3p targets among upregulated transcripts (10), suggesting that this miRNA could promote the pro- to pre-B transition. Interestingly, the about 50% smaller mutant pre-B population was sufficient to permit normal accumulation of immature and transitional B cells in Mb1-cre^+^ miR-142^fl^ mice (**Figures 1**, **2**). These observations agree with the capacity of as little as a third of typical pre-B cell numbers to fully populate the splenic B compartment (53).

The participation of the miR-142 locus in B cell physiology was more obvious in the mature B compartment of Mb1-cre^+^ miR-142^fl^ mice, consistent with the analyses of the miR-142-null animals. Both mutant models show that this locus is necessary for the generation of B1 cells and, in particular, for the regulation of CD23 levels on B cells (**Supplementary Figures 1**, **2**) (17). The presence of a substantial CD23^lo/–^ mutant mature B subset begs the question of its identity. On the one hand, concurring with the work of Kramer et al. (17), these mutant cells display MZ B cell features such as a high sensitivity to S1P chemotactic signals and a substantial fraction of the population is CD62L^lo/–^ (**Figure 7** and **Supplementary Figure 5**). On the other hand, the mutant mature B cells share characteristics with wild-type Fo B cells, including their expression levels of IgD and LFA-1 as well as their responses to homing chemokines (**Figures 2**, **4**). In the spleen, miR-142-deficient B cell positioning in the periphery of the white pulp is also unlike typical MZ B cells (**Figure 2**). The latter could instead reflect the localization of abnormal Fo B cells outside of the white pulp. Indeed, the ectopic expression of S1PR1 leads to the accumulation of Fo B cells in the red pulp *in vivo*, and enhanced Itgβ1 could prolong the mutant B cell residence in the marginal zone, by strengthening their interaction with VCAM-1 present in that zone (40, 54). While the identity of the CD23^lo/–^ mutant mature B cells remains to be clarified, there was nevertheless a striking difference in the numbers of splenic B cells between the two mutant mice, with a slight reduction in Mb1-cre^+^ miR-142^fl^ and a strong accumulation in miR-142^–/–^ animals (**Figure 2** and **Supplementary Figure 3**) (17, 19). In the latter situation, increased cell survival downstream of enhanced BAFFR expression (17), and possibly improved B cell production (**Supplementary Figure 3**), support the higher mature B cell numbers. The enlarged progenitor pool in miR-142-null mice may be a consequence of aberrant BAFFR expression too, since the elimination of one copy of this receptor limits the accumulation of miR-142-deficient splenic B cells (17). Thus, either BAFFR levels are not increased on B cells from Mb1-cre^+^ miR-142^fl^ mice or more likely a B cell extrinsic perturbation of the immune system is playing a role in the accumulation of B cells in the knockout animals. Overexpression of BAFFR ligand (i.e. BAFF), leads to the expansion of mature B cells (55). The enlarged neutrophil population (**Supplementary Figure 3**) (16, 17, 19), which can produce BAFF (56), could possibly boost the availability of the latter cytokine in miR-142^-/-^ mice.

In addition, the specific reduction of LN B cellularity in naïve Mb1-cre^+^ miR-142^fl^ mice (**Figure 3**) allowed us to identify an as yet unrecognized role for the miR-142 locus in the establishment of the migratory properties of mature B cells, more precisely recirculating Fo B cells. One mechanism influenced by the miR-142 miRNAs seems to be the entry of B cells into LNs as highlighted by the limited accumulation of mutant B cells in these tissues 2 hours after adoptive transfer and the impaired transcription of CD62L (**Figures 5**, **6**) (5, 41–43). The expression of KLF2 and FOXO1, two regulators of CD62L production (44–47), are moderately reduced in the splenic miR-142-deficient B population (**Figure 5**). Although FOXO1 appears more important than KLF2 for the accumulation of LN B cells (44, 46, 47), low KLF2 levels could further limit Sell transcription in the context of reduced FOXO1 in the mutant B cells. The weakening of Foxo1 transcription does not appear to be due to elevated expression of the predicted miR-142-5p target BCOR (**Figure 5**), a component of the polycomb repressive complex 1.1 active at the Foxo1 locus (48). However, we cannot exclude that miR-142-5p does not affect *Bcor* stability but exclusively represses its translation, as reported for other miRNAs (57, 58). Remarkably, FOXO1 protein levels were proportionally more affected than its transcript levels in splenic mutant B cells (**Figure 5**), suggesting that miR-142 miRNAs are more critical in limiting FOXO1 degradation. The activity of FOXO1 is regulated by the PI3K – Akt axis, including *via* triggering its proteolysis (59, 60). The miR-142 locus has been suggested to restrict the activation of the Akt kinase in the context of BAFF and LIF signaling in B and embryonic stem cells, respectively (17, 61). In the latter cells, the expression of KRAS, which can act upstream of Akt in signaling, has been reported to be directly controlled by both miR-142-5p and -3p (61). Although stimulated KRAS-deficient mature B cells appear to activate Akt (62), consistent with functional redundancy between Ras homologs, it is possible that an abnormal accumulation of KRAS in the mutant mature B cells could aberrantly boost Akt activity. Absence of miR-142 miRNAs could sensitize Fo B cells to environmental cues leading to increased activity of Akt and hence reduced FOXO1, consistent with roles for miRNAs in placing thresholds on cell signaling (12, 63).

These miRNAs may not only permit the entry of B cells into the LNs but also slow down their exit. Indeed, miR-142-deficient Fo B cells showed improved migration *in vitro* in response to S1P (**Figure 7**). S1P signals are transduced in B cells through S1PR1 and -3, which support their egress *in vivo* and chemotaxis *in vitro*, respectively (6, 8, 28, 37). Notably, mutant mature B cells demonstrated higher abundance of surface S1PR1 in the spleen and LNs, compared to controls (**Figure 7**). The ablation of the miR-142 locus did not lead to obviously higher transcript levels of S1PR1 and S1PR3 in splenic mutant B cells (**Figure 7**). These results also imply that miR-142 miRNAs do not regulate the mRNA stability of these two predicted targets, although an unusual effect on solely the translation of *S1prs* cannot be ruled out. Expression of the GRK2 kinase, which limits the accumulation of S1PR1 on B cells (50, 51), appears only mildly affected and hence likely does not significantly perturb the biology of S1PR1 in mutant B cells (**Figure 7**). Thus, further investigations are needed to show a role for miR-142 miRNAs in modulating S1P-mediated B cell egress from LNs *in vivo* and identify the mechanism regulating S1PR1 expression. Inactivation of the miR-142 locus in B cells could also improve S1P receptor signaling. Akt could be a kinase of interest in this context since it is downstream of both S1PRs and its activity is (indirectly) modulated by miRNAs from the miR-142 locus as discussed above (17, 61, 64, 65).

In general, the present work underscores the several important cell autonomous roles of the miR-142 locus in the B cells of naïve mice. Thus, this locus modulates the size of the pre-B compartment and is essential for the generation of a proper mature B cell compartment. miR-142 miRNAs appear also key for establishing optimal mature B cell migratory properties, modulating the capacity of Fo B cells to patrol the organism and monitor for the presence of their cognate antigens.

## Data availability statement

The original contributions presented in the study are included in the article/**Supplementary Material**. Further inquiries can be directed to the corresponding author.

## Ethics statement

The animal study was reviewed and approved by the Bundesministerium für Bildung, Wissenschaft und Forschung (Austria) and the Landesamt für Gesundheit und Soziales Berlin (Germany).

## Author contributions

MHa, TC, VL, KR and ED designed the research. TC generated the miR-142^fl^ allele. MHa, WO, MHe, NHK, JK, BJ, JP, VL and ED performed the experiments. MHa, WO, BJ, KR and ED analyzed the data. ED wrote the manuscript with contributions from MHa, WO and MHe. All authors contributed to the article and approved the submitted version.

## Funding

ED was supported by funding from the Nachwuchsförderung der LFU (2017/BIO-5), the Tiroler Wissenschaftsförderung (UNI-0404/2310) and an Aktion D. Swarovski KG (2016/BIO-20). VL was supported by the FWF (Austrian Science Fund) grant P32755. MHa was supported by funding from the Exzellenzstipendien für Doktoratskollegs (DK) der LFU (2020/BIO-40).

## Acknowledgments

We thank J. Cernoch, C. Salomon, W. Kapferer and B. Szalka for technical assistance; J. Kunert, A. Ullmann, X. Richter, N. Heinrich and W. Plunger for help with animal care; P. Jansen-Dürr for scientific discussions; R. Lauhkonen-Seitz, D. Ram, J. Krabichler, A. Hohenegger, E. Schweinberger and G. Guem for administrative and IT assistance.

## Conflict of interest

TC is currently employed by Vor Biopharma; however, the work by this author was done exclusively at the first affiliation (Harvard Medical School).

The remaining authors declare that the research was conducted in the absence of any commercial or financial relationships that could be construed as a potential conflict of interest.

The handling editor DRW declared a shared parent affiliation with the authors TC, KR, at the time of review.

## Publisher’s note

All claims expressed in this article are solely those of the authors and do not necessarily represent those of their affiliated organizations, or those of the publisher, the editors and the reviewers. Any product that may be evaluated in this article, or claim that may be made by its manufacturer, is not guaranteed or endorsed by the publisher.

## Supplementary material

Supplementary Figure 1 Increased frequency of CD23^lo/–^ follicular B cells in Mb1-cre^+^ miR-142^fl^ mice. Left: FACS analysis of CD23^+^ and CD23^lo/–^ cells within **(A)** splenic (Spl) CD19^+^B220^+^CD93^–^CD21^+^IgM^+^CD1d^+^ follicular B (FoB) cells, as well as within **(B)** lymph nodes (LN) and **(C)** bone marrow (BM) CD19^+^B220^+^CD93^–^IgM^+^CD21^+^ Fo B cells of Mb1-cre^+^ control and Mb1-cre^+^ miR-142^fl^ mice. Center: Proportions of CD23^lo/–^ cells within Fo B cells in **(A)** Spl, **(B)** LN and **(C)** BM of Mb1-cre^–^ (□), Mb1-cre^+^ (○) and Mb1-cre^+^ miR-142^fl^ (▲) mice. *n* = 3-13 per group, cumulative of 6-14 independent experiments. Right: Geometric means (Geo. mean) of surface levels of CD23 on CD23^+^ Fo B cells and CD21 on Fo B cells in **(A)** Spl and **(B)** LN of the above groups of mice. *n* = 3-6 per group in 6 independent experiments. **(A–C)** Each symbol indicates one mouse. Horizontal blue lines signify the means. **, *P* ≤ 0.01; ****, *P* ≤ 0.0001 by one-way ANOVA.

Supplementary Figure 2 Mb1-cre^+^ miR-142^fl^ mice lack peritoneal cavity B1 cells. **(A)** FACS analysis of CD19^+^B220^+/–^ B cells within lymphocytes as well as CD19^+^B220^+^ B2 and CD19^+^B220^lo/–^ B1 cells within B cells in the peritoneal cavity of Mb1-cre^+^ control and Mb1-cre^+^ miR-142^fl^ mice. **(B)** Frequency of B1 cells within the peritoneal cavity B cells of Mb1-cre^–^ (□), Mb1-cre^+^ (○) and Mb1-cre^+^ miR-142^fl^ (▲) mice. *n* = 5-9 per group in 10 independent experiments. Each symbol represents one mouse. Horizontal blue lines indicate the means. ***, *P* ≤ 0.001 by one-way ANOVA.

Supplementary Figure 3 Constitutive deletion of miR-142 leads to an aberrant immune system. **(A)** Top: Flow cytometry of CD19^+^B220^+^ B cells as well as IgM^+^CD1d^+^ follicular and IgM^hi^CD1d^hi^ marginal zone B cells within CD93^–^ mature B cells in the spleens of wild-type and miR-142^–/–^ mice. Bottom: Splenic B and follicular B (FoB) cell numbers in the spleens of wild-type (□) and miR-142^–/–^ (◆) animals. *n* = 15-17 per group in 14-16 independent experiments. **(B)** Top: FACS analysis of CD93^+^ progenitor B cells in CD19^+^B220^+^ bone marrow B cells and CD25^+^ pre-B cells within IgM^–^c-Kit^–^ progenitor B cells, as well as B220^lo^IgM^–^ progenitor B cells in CD19^+^B220^+^ splenic B cells and CD25^+^ pre-B cells within CD93^+^c-Kit^–^ progenitor B cells in wild-type and miR-142^–/–^ mice. Bottom: IgM^–^c-Kit^+^ pro-, IgM^–^CD25^+^ pre- and IgM^+^ immature (Imm.) B cell numbers in the bone marrow (BM) and spleens (Spl) of wild-type (□) and miR-142^–/–^ (◆) animals. *n* = 6-16 per group, pooled from 6-15 independent experiments. **(C)** TCRβ^+^CD3ε^+^ T cell numbers and **(D)** CD11b^+^Ly6G^+^ neutrophil cellularity in the spleens of control and mutant mice. *n* = 13-16 per group, cumulative of 13-15 independent experiments. **(A–D)** Each symbol depicts one mouse. Horizontal blue lines signify the means. *, *P* ≤ 0.05; **, *P* ≤ 0.01; ***, *P* ≤ 0.001; ****, *P* ≤ 0.0001 by Student’s *t* test **(A–D)** and one-way ANOVA **(B)**. **(C)** Sample excluded from the analysis: One wild-type mouse for an aberrantly high value.

Supplementary Figure 4 Strategy to identify splenic follicular and marginal zone B cells in transwell migration assay *in vitro*. Flow cytometry of control (Mb1-cre^+^) and mutant (Mb1-cre^+^ miR-142^fl^) IgM^+^CD1d^+^ follicular as well as IgM^hi^CD1d^hi^ marginal zone B cells within CD19^+^B220^+^CD93^–^ mature B cells in the input (splenocytes, without transwell) and in the bottom chamber of the transwell in absence of chemoattractants (no treatment) as well as upon treatment for 3h with 1 μg/ml CCL21, 100 ng/ml CXCL12 and 100 nM S1P, for example. Data were acquired for a 90s period.

Supplementary Figure 5 Reduced CD62L expression is not restricted to the CD23^lo/–^ follicular B cells of Mb1-cre^+^ miR-142^fl^ mice. **(A)** Geometric means (Geo. mean) of CD62L surface levels on CD19^+^B220^+^CD93^–^IgM^+^CD21^+^CD62L^+^ Fo B cells in the spleens (Spl) and lymph nodes (LN) of Mb1-cre^+^ (○) as well as Mb1-cre^+^ miR-142^fl^ (▲) mice, as determined by flow cytometry. *n* = 3-4 per group in 4 independent experiments. **(B)** Proportions of CD62L^+^ cells within CD23^+^ (black) and CD23^lo/–^ (grey) IgM^+^CD21^+^ Fo B cells in Spl and LNs of Mb1-cre^+^ (○) as well as Mb1-cre^+^ miR-142^fl^ (▲) mice. *n* = 7-8 per group, cumulative of 8 independent experiments. **(A, B)** Each symbol depicts one mouse. Means are indicated by horizontal blue lines. *, *P* ≤ 0.05; **, *P* ≤ 0.01; ****, *P* ≤ 0.0001 by Student’s *t* test **(A)** and one-way ANOVA **(B)**.

Supplementary Figure 6 Expression of FOXO1 and HSP90 in magnetically-purified control and miR-142-deficient splenic B cells. Complete images of the chemoluminescent signals recorded for the Western blotting of (top) FOXO1 and (bottom) HSP90 depicted in **Figure 5**. Positions of the molecular weight standards (in kDa) were inserted electronically on the left.
