# Supplementary material for: miR-142 favors naïve B cell residence in peripheral lymph nodes
Source: Front Immunol. 2022 Nov 10;13:847415. doi: 10.3389/fimmu.2022.847415 (PMC9686386; doi:10.3389/fimmu.2022.847415)
Supplement: Supplementary Figure 1 — Increased frequency of CD23lo/– follicular B cells in Mb1-cre+ miR-142fl mice. Left: FACS analysis of CD23+ and CD23lo/– cells within (A) splenic (Spl) CD19+B220+CD93–CD21+IgM+CD1d+ follicular B (FoB) cells, as well as within (B) lymph nodes (LN) and (C) bone marrow (BM) CD19+B220+CD93–IgM+CD21+ Fo B cells of Mb1-cre+ control and Mb1-cre+ miR-142fl mice. Center: Proportions of CD23lo/– cells within Fo B cells in (A) Spl, (B) LN and (C) BM of Mb1-cre– (□), Mb1-cre+ (○) and Mb1-cre+ miR-142fl (▲) mice. n = 3-13 per group, cumulative of 6-14 independent experiments. Right: Geometric means (Geo. mean) of surface levels of CD23 on CD23+ Fo B cells and CD21 on Fo B cells in (A) Spl and (B) LN of the above groups of mice. n = 3-6 per group in 6 independent experiments. (A–C) Each symbol indicates one mouse. Horizontal blue lines signify the means. **, P ≤ 0.01; ****, P ≤ 0.0001 by one-way ANOVA. [file DataSheet_1.pdf]

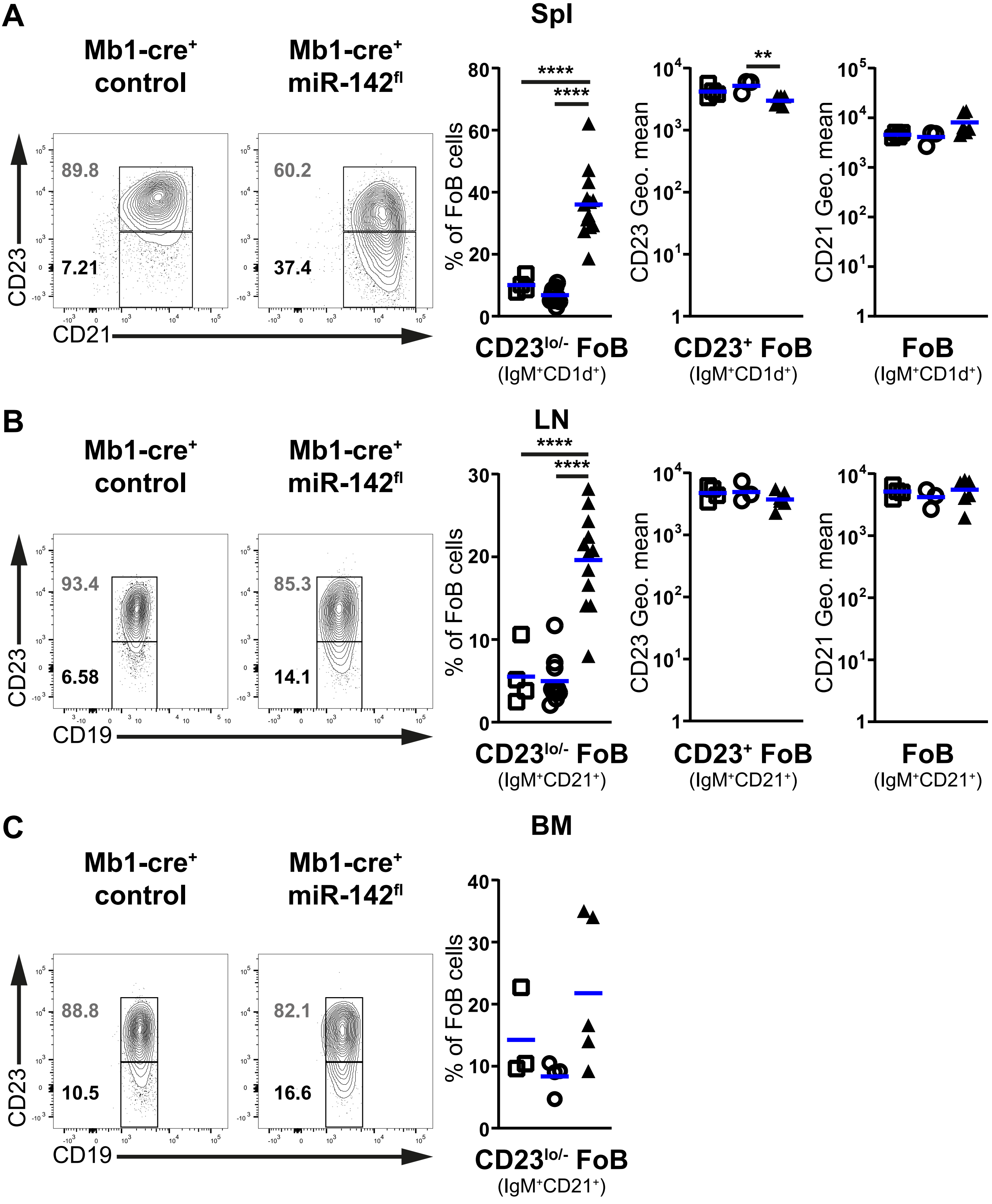

Supplementary Figure 1

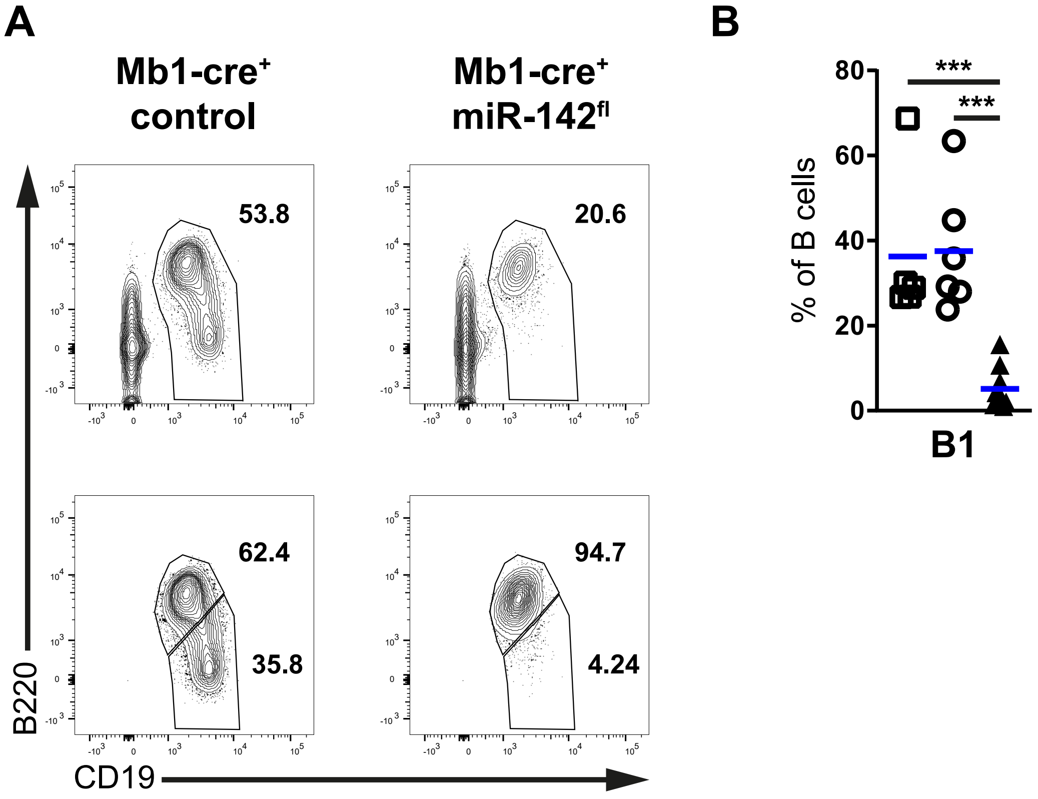

Supplementary Figure 2

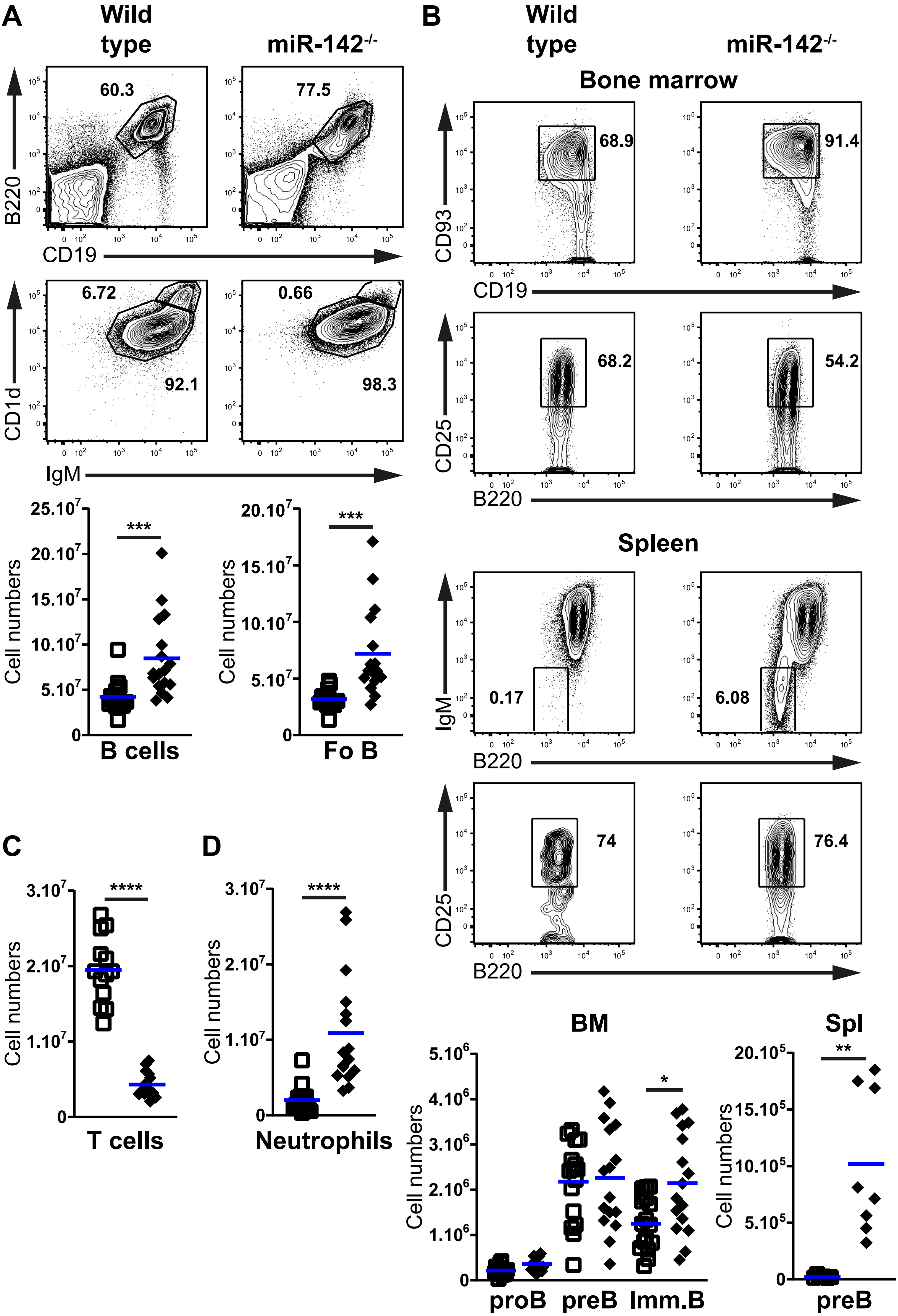

Supplementary Figure 3

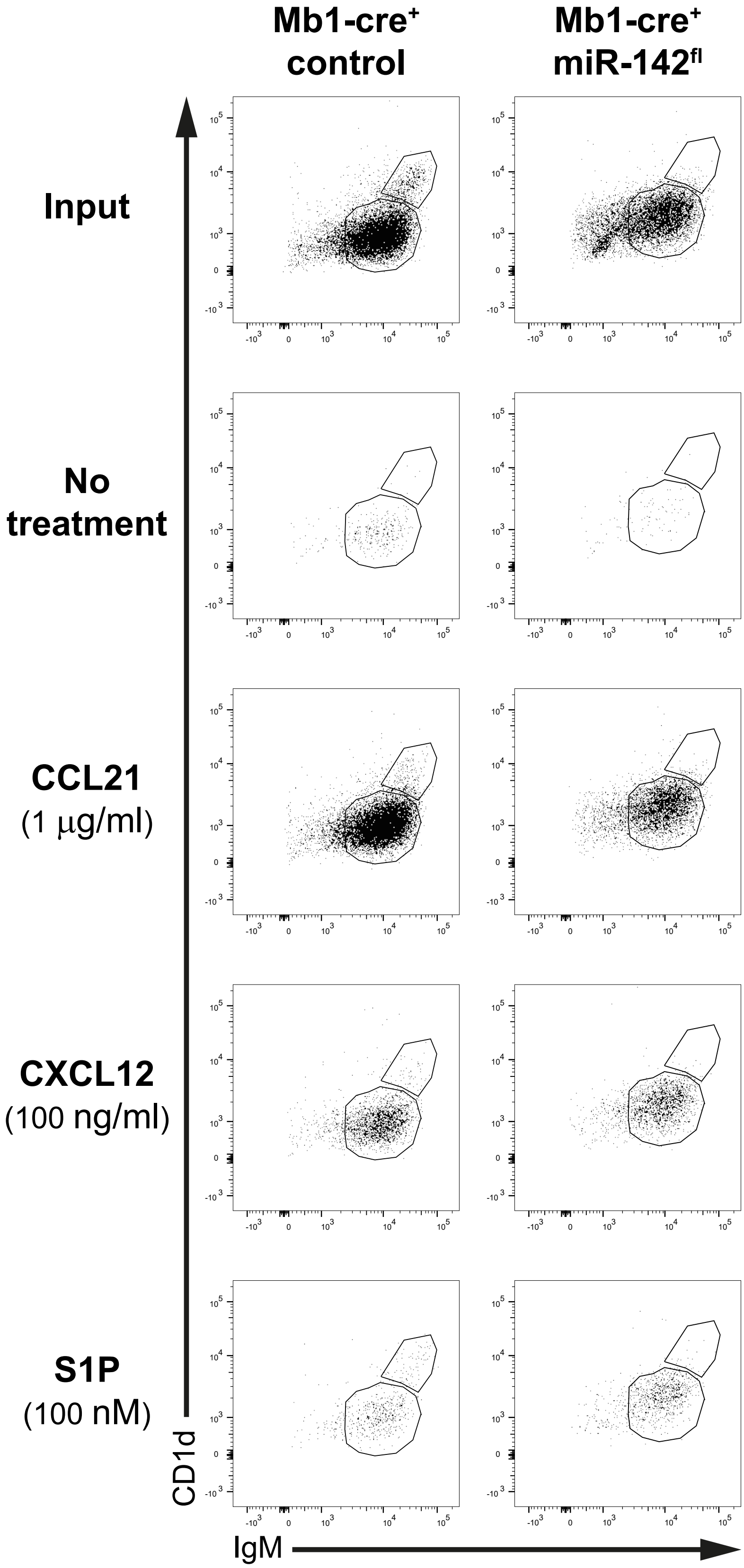

Supplementary Figure 4

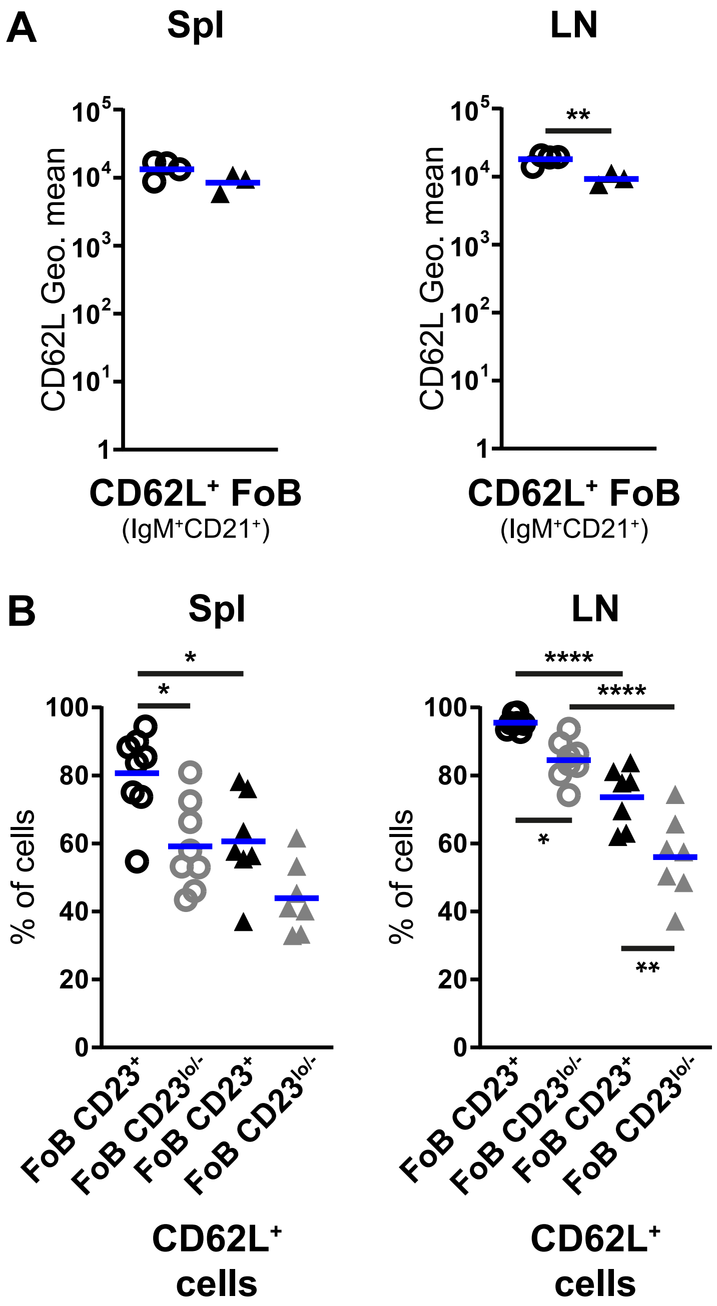

Supplementary Figure 5

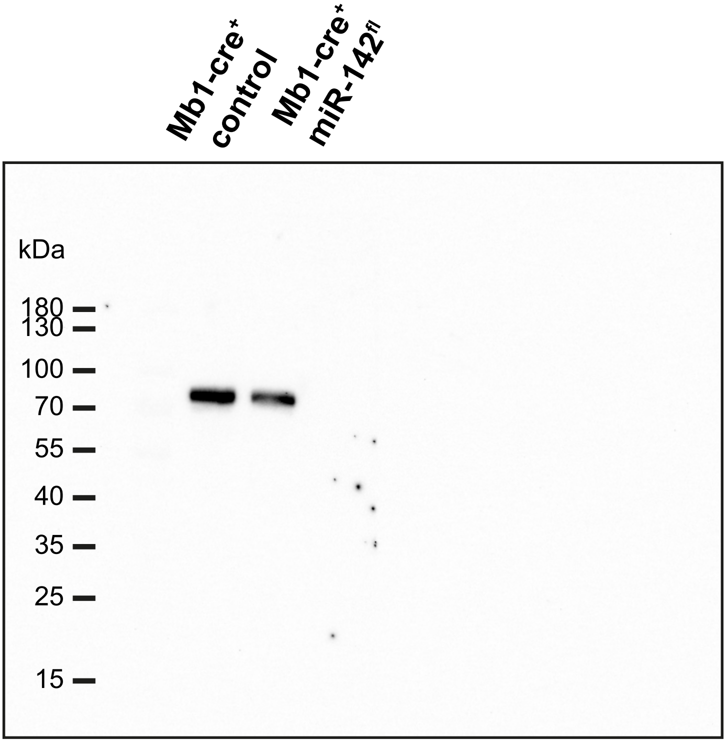

FOXO1

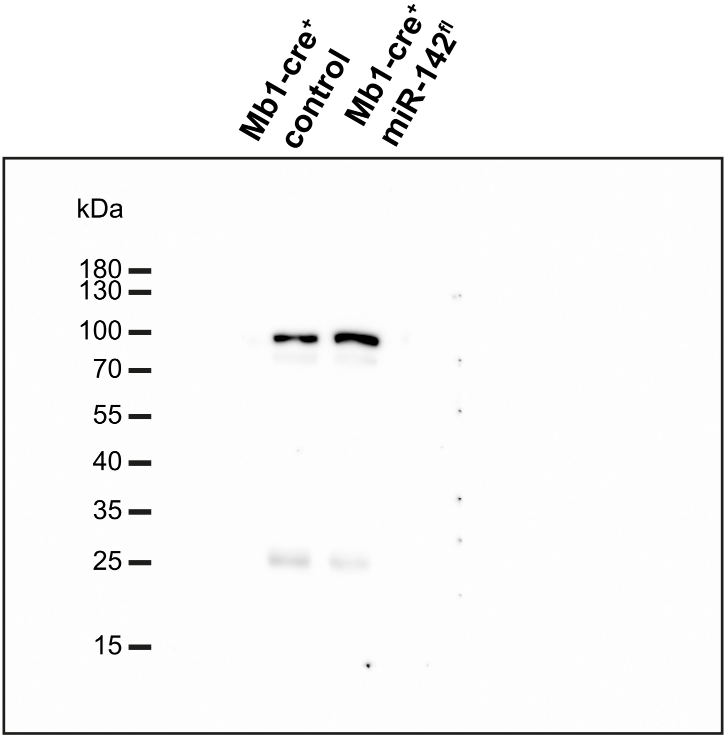

HSP90

Supplementary Figure 6
